# Consistency of serial ultrasonographic joint tissue measurements by the Joint _tissue_Activity and Damage Exam (JADE) protocol in relation to hemophilic joint health parameters

**DOI:** 10.1186/s12891-023-06419-5

**Published:** 2023-04-15

**Authors:** Richard F. W. Barnes, Peter Aguero, Cris Hanacek, Andres Flores, Bruno Steiner, Cindy Bailey, Doris V. Quon, Rebecca Kruse-Jarres, Annette von Drygalski

**Affiliations:** 1 Department of Medicine, Division of Hematology/Oncology, University of California San Diego, 9333 Genesee Avenue, St 310, San Diego, CA 92122 USA; 2 Department of Physical Medicine and Rehabilitation, University of California San Diego, San Diego, CA USA; 3 Washington Center for Bleeding Disorders, Seattle, WA USA; 4 Orthopaedic Hemophilia Treatment Center at Orthopaedic Institute for Children Los Angeles, Los Angeles, CA USA

**Keywords:** Hemophilia, Arthropathy, Hemarthrosis, Ultrasound, JADE, MSKUS

## Abstract

**Objectives:**

The Joint _tissue_Activity and Damage Exam (JADE) is a point-of-care (POC) musculoskeletal ultrasound (MSKUS) protocol for non-radiologists to evaluate hemophilic arthopathy. Our aim was to determine the consistency of cross-sectional analyses of direct tissue measurements (JADE protocol) and clinical Hemophilia Joint Health Score [HJHS] and functional joint assessments (arc) at three clinic visits.

**Methods:**

We prospectively studied adults (n = 44) with hemophilia (A or B) of any severity and arthropathy at 3 North American sites. We assessed HJHS, total arc, and JADE parameters (bilateral elbows, ankles, and knees) at study entry, at ≈12–18 months, and at ≈24–36 months, and used MSKUS to evaluate painful episodes between study visits. JADE measurements included osteochondral alterations, cartilage thickness, and soft tissue expansion at sentinel positions. Associations between joint HJHS and total arc with each JADE variable were examined with random intercept models.

**Results:**

At each visit increasing HJHS and decreasing total arc were associated in the expected direction with increasing length of OAs and soft tissue expansion in all joints, and decreasing cartilage thickness in the knee. However, HJHS associations with cartilage thickness were U-shaped for elbow and ankle (i.e. cartilage thinning and thickening). Associations between total arc and cartilage thickness followed a similar curve. (Near) normal levels of both joint parameters (HJHS and total arc) were associated with normal ranges of cartilage thickness. JADE views were also helpful to detect hemarthrosis in association with joint pains.

**Conclusions:**

POC MSKUS applying direct tissue measurements using the JADE protocol provided reproducible cross-sectional associations with joint health outcomes on three visits. These findings advance protocol validation and enable iterative adaptations resulting in JADE protocol version 2.

**Supplementary Information:**

The online version contains supplementary material available at 10.1186/s12891-023-06419-5.

## Introduction

The Joint tissue Activity and Damage Exam (JADE) protocol was developed to provide point-of-care (POC) imaging with musculoskeletal ultrasound (MSKUS) for non-radiologists to evaluate the extent and progression of hemophilic arthropathy in the ambulatory setting [1]. The JADE protocol uses quantitative numerical measurements (1/10^th^ of a millimeter tissue resolution) for osteochondral alterations, cartilage thickness, and soft tissue expansion in sentinel positions of the elbow, knee, and ankle [1]. The validation of the JADE protocol has followed processes recommended by the international Outcomes Measures in Rheumatology (OMERACT) guidelines (“truth, discrimination, feasibility” [2]) to ensure pathological tissue recognition in hemophilic arthropathy [3], high intra/inter-rater and inter-operator reliability [1], and cross-sectional associations with clinical parameters at baseline [4].

We previously reported that joint tissue measurements according to the JADE protocol were associated with clinical (Hemophilia Joint Health Score [HJHS]) and functional (total arc) joint assessments at baseline for persons with hemophilia (PWH) who were afflicted by a broad spectrum of arthropathic manifestations. The previous effort was limited to one-time measurements in a cross-sectional cohort analysis [4]. Here we further evaluate the reliability of JADE measurements using the same cohort by evaluating the cross-sectional association of direct tissue measurements with clinical and functional joint assessments, performed sequentially at several clinic visits (≈1 year apart) during a prospective ≈3-year study period. These analyses were geared to determine the consistency of such associations for the JADE protocol views at different clinic visits over time following OMERACT guidelines [2], which recommend iterative validation efforts.

## Materials and methods

### Patient selection

Between May 2016 and April 2019, we recruited adult (age ≥ 18 years) PWH (Hemophilia A or B) irrespective of severity at three Hemophilia Treatment Centers in the United States (Los Angeles Orthopedic Hemophilia Treatment Center, University of California San Diego, and Washington Center for Bleeding Disorders). PWH were included if they had at least one arthropathic joint defined by HJHS, which was recently validated for use in adult PWH [5]. HJHSs had to have values that were at least three or higher, to suggest arthropathic changes [6]. Besides the absence of arthropathy by those criteria there were no other study exclusion criteria. Each patient made three visits to his Treatment Center during a period of approximately 2–3 years. The cross-sectional results obtained during each visit are reported here without including longitudinal analyses. The study was approved by the Institutional Review Boards of the participating institutions (Institutional Review Board #:120,510). Each patient and/or his legal guardian provided written informed consent for study participation. The study was performed in accordance with the Declaration of Helsinki.

### Joint health evaluation

Joint health of both elbows, knees, and ankles was assessed by HJHS, total arc, and POC MSKUS, at three time points (“visits”): study entry (baseline), at ≈12–18 months post (midpoint), and at ≈24–36 months post (final). The study visits were performed when patients were in their usual state of health. Subjects also were evaluated within a window of 48 h when experiencing acute joint pains. We used HJHS version 2.1 (HJHS per joint: 0 best, 20 worst; total HJHS for 6 joints combined: 0 best, 120 worst [6]). The HJHS version 2.1 is an established outcome measure, providing a clinical score for each joint summarizing swelling, duration of swelling, pain, strength, loss of range of motion (ROM), muscle atrophy, and crepitus. HJHSs were performed by a licensed physical therapist with > 5 years of general practice experience and approximately 2 years of experience with hemophilia patients (BS, PA, CB). The physical therapists were trained in the HJHS acquisition according to instructions and guidance provided by online training and video modules developed by the International Prophylaxis Study Group (http://www.ipsg.ca/publication/hemophilia-joint-health-score-instructional-video-and-manual). Total arc, a functional assessment of the sum of flexion and extension degrees of range of motion (ROM), was measured by goniometry [7–9]. Higher HJHS and lower total arc measurements reflect worse joint health.

### POC MSKUS imaging

POC MSKUS measurements were made with a GE Logiq S8 high-resolution ultrasound machine (General Electrics, Fairfield, Connecticut) with real-time spatial compound imaging, an 8–15 MHz high-frequency grayscale (B-mode) linear transducer, and speckle reduction capabilities in accordance with the manufacturers. At each participating institution, images were obtained by hemophilia providers with at least 3 years of experience and formally trained in POC MSKUS in the UCSD CME-accredited course (https://cme.ucsd.edu/httc/index.html). Two providers (AvD and BS) are certified in MSKUS by the Alliance for Physician Certification & Advancement (APCA), and PA and CB were in the process of certification (PA now certified). Images were captured by the JADE protocol version 1 [1]. Operators were the same during all visits at the Los Angeles Orthopedic Hemophilia Treatment Center (CB) and the Washington Center for Bleeding Disorders (BS). There were two operators at the University of California San Diego (PA and AF). Briefly, 21 standardized positions in the elbow, knee and ankle evaluated cartilage thickness, osteochondral alterations, and soft tissue expansion. Cartilage thickness and osteochondral alterations were measured on the elbow humeral capitulum in short and long axis, patellofemoral knee joint space in short axis, long axis on the medial and lateral femoral condyles posterior and anterior, and the ankle talar dome (short axis for osteochondral alterations and long axis for cartilage thickness). Soft tissue expansion was measured at the elbow olecranon fossa, knee medial and lateral recesses, and the ankle anterior recess of the tibiotalar joint. The soft tissue areas include fat pads, synovium, and capsular soft tissue structures measured as a conglomerate because they cannot be differentiated by POC MSKUS in pathological states [3]. Worse joint health is reflected by increased length of osteochondral alterations, decreased cartilage thickness, and/or increased soft tissue expansion. MSKUS evaluation during painful episodes included sonopalpation to evaluate compressibility and displacement of intra-articular material in the joint recesses to distinguish between simple and complex (bloody) effusions [10].

### Statistical analyses

The results are presented at the joint level (JADE): elbow, knee, and ankle. We examined two outcomes for each joint: joint HJHS and total arc. The HJHS values were not normally distributed, but rather positively skewed, therefore we applied a square root transformation, where *sqrt*HJHS = *√*(0.5 + HJHS). In contrast, the negative skew of the values for total arc was corrected with a reflecting transformation [11] where the transformed value *Y* = *ln*(170 – Total arc).

The three predictors were the JADE MSKUS measurements of length of osteochondral alteration, cartilage thickness, and soft tissue expansion. Values that were not normally distributed and might exert disproportionate leverage upon the estimates were log transformed before analysis. Values that were more than 3.29 standard deviations from the mean were assumed to be drawn from a different population (*P* < 0.001) [11]. They were classified as outliers and dropped from the analysis.

For each visit (baseline, midpoint and final), we plotted graphs and calculated the best fit curve for the association between each JADE variable predictor and the two outcomes. Because each subject had left and right joints, we adjusted for the intra-individual correlation by fitting random intercept models [12] to calculate the line that best described the association between predictors and outcomes. Curves were fitted by adding polynomial terms. As previously described by Mesleh Shayeb et al. [4], the association between the outcome and JADE variable was shown by the regression coefficient *b* and 95% confidence interval. The use of a reflecting transformation for total arc means that a negative regression coefficient indicates a positive association between the outcome and the independent variable, and vice-versa [4]. With a quadratic model the association was described by two regression coefficients *b*_*1*_ and *b*_*2*_ [4]. We plotted the curves for baseline, midpoint, and final visits on the same graph. If tests for parallelism and equal intercepts both showed no evidence for a difference, then we concluded that the three curves coincided [13]. Descriptive statistics were applied for the assessment of acute painful joint episodes.

## Results

### Patient characteristics

Table 1 includes baseline characteristics of 44 study participants. Briefly, the median age was 36 years (Interquartile Range [IQR]: 28, 49). Most participants were White, had severe Hemophilia A, and received prophylactic factor replacement. The time between baseline and final visits ranged from 1 to 2.9 years with a median of 1.6 years (IQR: 1.3, 1.9). Three-quarters of the participants were followed for almost 2 years. None of the participants had experienced hemarthrosis or trauma within the 3 months preceding the evaluation visits.

**Table 1:** Baseline characteristics

**Characteristic**	
Center^a^	
LA	14 (32)
Seattle	15 (34)
San Diego	15 (34)
Hemophilia type^a^	
A	35 (80)
B	9 (20)
Severity^a^	
Mild	1 (2)
Moderate	7 (16)
Severe	36 (82)
Race/ethnicity^a^	
Hispanic	7 (16)
White	26 (59)
African American	4 (9)
Asian	2 (5)
Other	5 (11)
Age (years)^b^	36 [28, 49]
Total HJHS^b^	27 [18, 42]
Joint HJHS^b^	
Elbow	2 [0, 7]
Knee	2 [0, 7]
Ankle	6 [1, 9]
HJHS^b^	
Elbow: Left	2 [0, 7]
Right	2 [0, 7]
Knee: Left	2 [0, 7]
Right	2 [0, 8]
Ankle: Left	6 [1, 9]
Right	6 [2, 9]
Joint Total arcs^b^	
Elbow	130 [100, 143]
Knee	130 [105, 140]
Ankle	32 [22, 56]
Total arcs^b^	
Elbow: Left	130 [99, 143]
Right	130 [100, 143]
Knee: Left	131 [105, 140]
Right	130 [97, 140]
Ankle: Left	32 [22, 56]
Right	30 [22, 53]
Prophylaxis	
Regular prophylaxis	39
On demand	3
Gene therapy	2
Joints replaced	
Ankle	1
Elbow	1
Knee	18
Joints fused	
Ankle	6
Elbow	0
Knee	0

*HJHS* Hemophilia Joint Health Score

^a^values expressed as N (%)

^b^values expressed as median [interquartile range]

### JADE POC MSKUS measurement associations with HJHS and total arc

#### Effect of transformations

Transforming the overall bodily HJHS values improved the distribution slightly in terms of a better fit to normal (Kolmogorov–Smirnov two-sample test: D = 0.117, *p* = 0.130 for HJHS; D = 0.095, *p* > 0.150 when transformed). Comparing the fit to a normal distribution at the joint level showed that transformation of HJHS resulted in a small improvement for the knee; transforming total arc slightly improved the fit for elbow, knee and ankle (Table S1a). The effect on the models is illustrated by examining the residuals and standard errors. Transformations of HJHS brought small improvements for the model’s residuals for the elbow and ankle, but not for the knee (Table S1b). Transformation of total arc slightly improved residuals for the elbow and knee, but not for the ankle. The models with residuals closer to normal produced regression estimates with smaller standard errors (Table S1c) and therefore narrower confidence intervals. In summary, transformation resulted in more precise estimates for two-thirds of the models.

#### Elbow

**Fig. 1:**
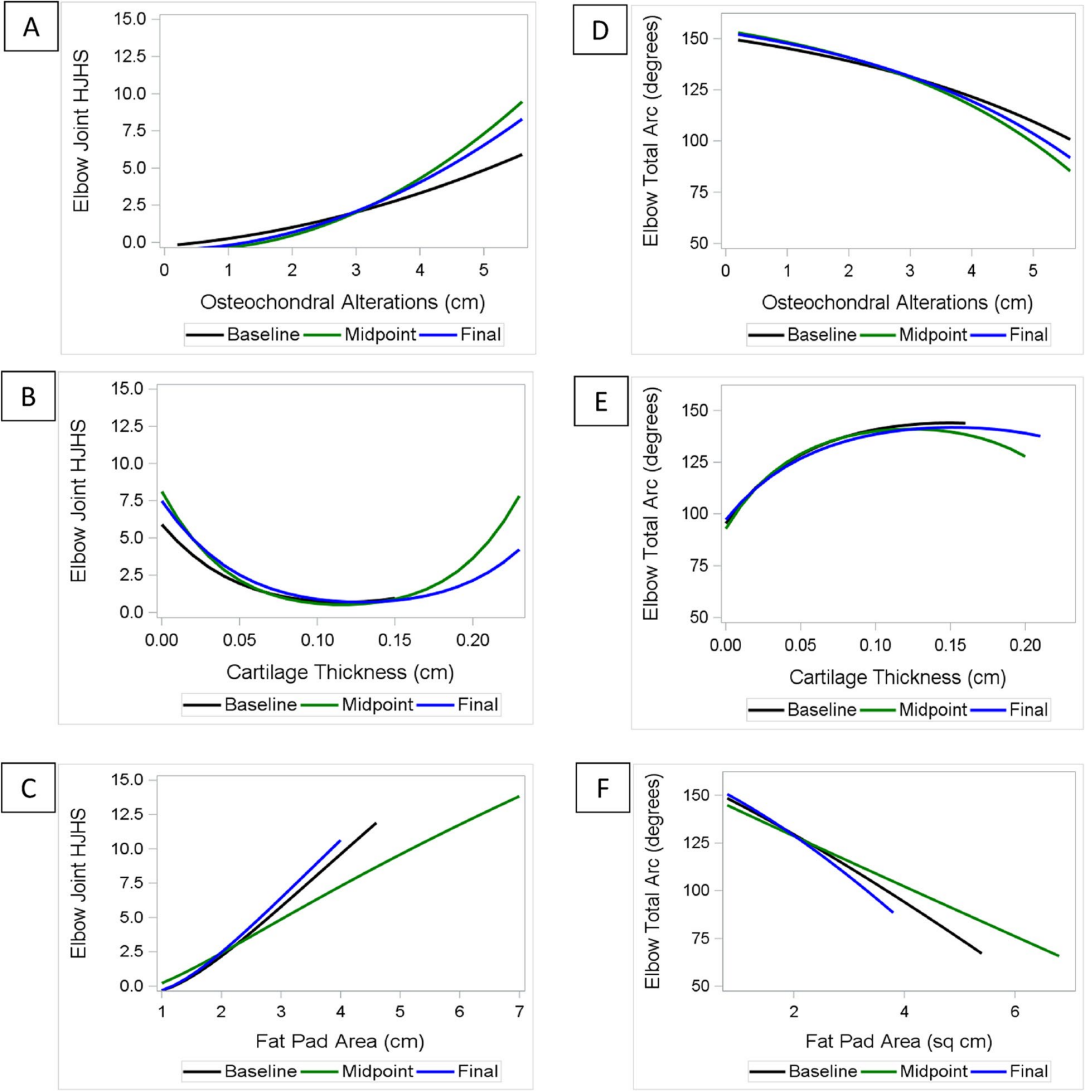
Elbow: Association of Joint _Tissue_Activity and Damage Exam variables with HJHS and total arc at each time point. Each patient had three time points (initial, midpoint, and final clinic visit). At each time point, the association is represented between HJHSs and JADE variables: **a** length of osteochondral alterations; **b** cartilage thickness; and **c** and soft tissue expansion of the olecranon recess fat pad. At each time point, the association is represented between total arcs and JADE variables: **d** Length of osteochondral alterations; **e** cartilage thickness; and **f** soft tissue expansion of the olecranon recess fat pad. The regression equation for each plot is shown in Table S2. JADE: Joint _Tissue_Activity and Damage Exam; HJHS: Hemophilia Joint Health Score

At each of the three visits there was a positive association between HJHS with increasing length of osteochondral alterations on the humeral capitulum and soft tissue expansion in the olecranon fossa (Fig. 1a and 1c, Supplement 1: Table S2). Regarding humeral capitulum cartilage thickness, the HJHS was lowest (≈0–2) at a cartilage thickness of ≈0.1–0.15 cm (normal range reported between 0.04–0.18 [14]) but increased when the cartilage thickness was either lower than 0.1 or higher than 0.15 cm (Fig. 1b).

Changes in total arc followed a similar pattern. At each visit declining total arcs were associated with increasing osteochondral alterations and greater soft tissue expansion (Fig. 1d and 1f, Supplement 1: Table S3). Also, like HJHS, the total arc was highest at a humeral capitulum cartilage thickness of ≈0.1–0.15 cm and declined with thinning of the cartilage (Fig. 1e).

#### Knee

**Fig. 2:**
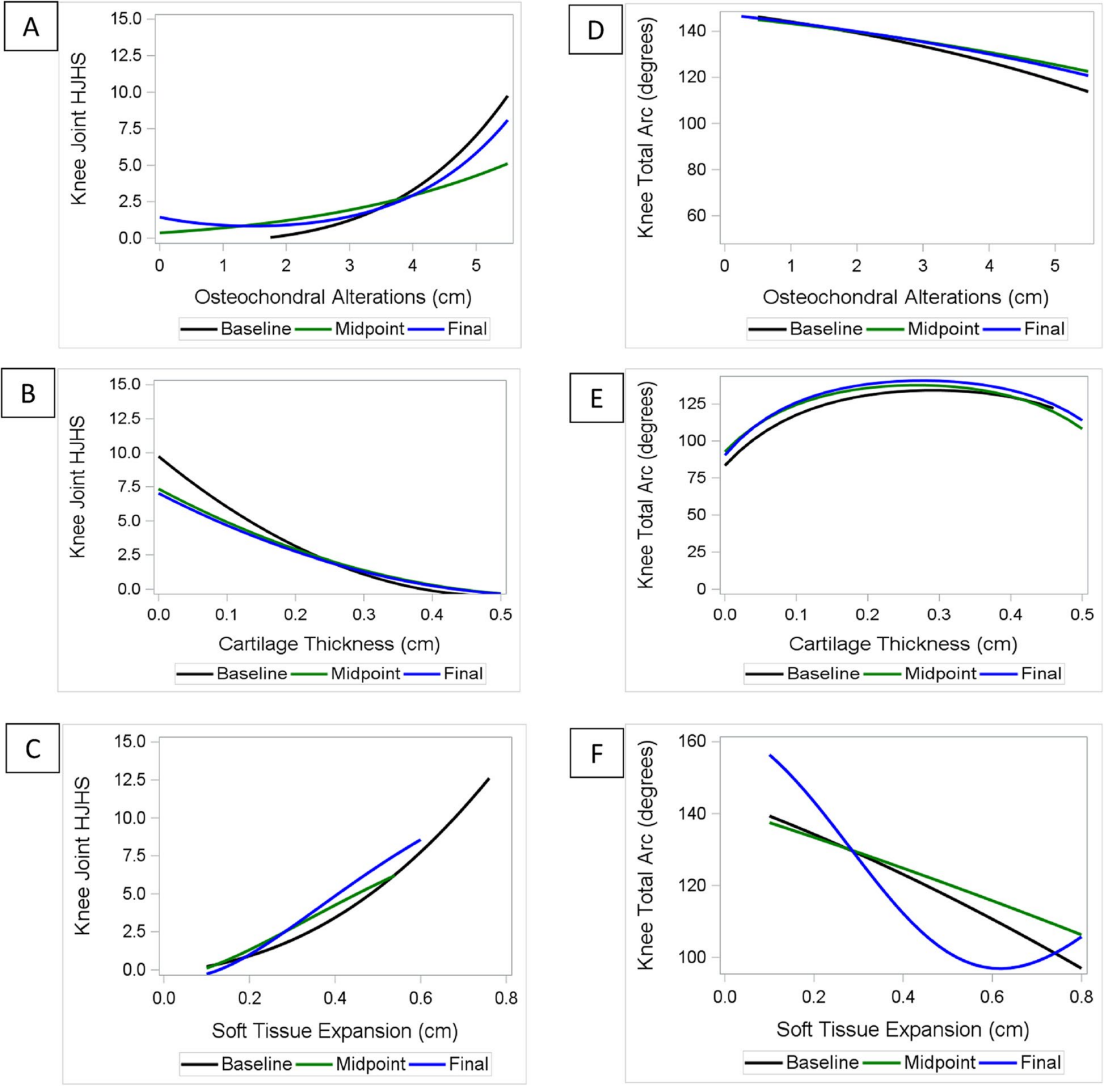
Knee: Association of Joint _Tissue_Activity and Damage Exam variables with HJHS and total arc at each time point. Each patient had three time points (initial, midpoint, and final clinic visit). At each time point, the association is represented between HJHSs and JADE variables: **a** length of osteochondral alterations; **b** cartilage thickness; and **c** soft tissue expansion of the average of lateral and medial recess. At each time point, the association is represented between total arcs and JADE variables: **d** length of osteochondral alterations; **e** cartilage thickness; and **f** soft tissue expansion of the average of lateral and medial recess. The regression equation for each plot is shown in Table S3. JADE: Joint _Tissue_Activity and Damage Exam; HJHS: Hemophilia Joint Health Score

At each of the three visits there were positive associations between HJHS with increasing length of osteochondral alterations in the patellofemoral joint space, and with greater soft tissue expansion in the medial and lateral recesses (Fig. 2a and 2c, Supplement 1: Table S4). Increasing HJHS was also associated with decreasing cartilage thickness in the patellofemoral joint space, following a relatively linear trajectory (Fig. 2b).

Changes in total arc followed a similar pattern demonstrating near linear associations of total arc with osteochondral alterations and soft tissue expansion (Fig. 2d and 2f, Supplement 1: Table S5). However, an inverted U-shape was apparent for cartilage thickness, whereby the values for the total arc were highest when cartilage thickness was within normal range (0.2 to 0.3 cm [15]) but decreased when cartilage thickness was below 0.2 cm or above 0.3 cm (Fig. 2e).

There were no associations noted for HJHS or total arc when measurements of osteochondral alterations and cartilage thickness were performed in long axis on the anterior and posterior femoral condyles, medial or lateral (results not shown).

#### Ankle

**Fig. 3:**
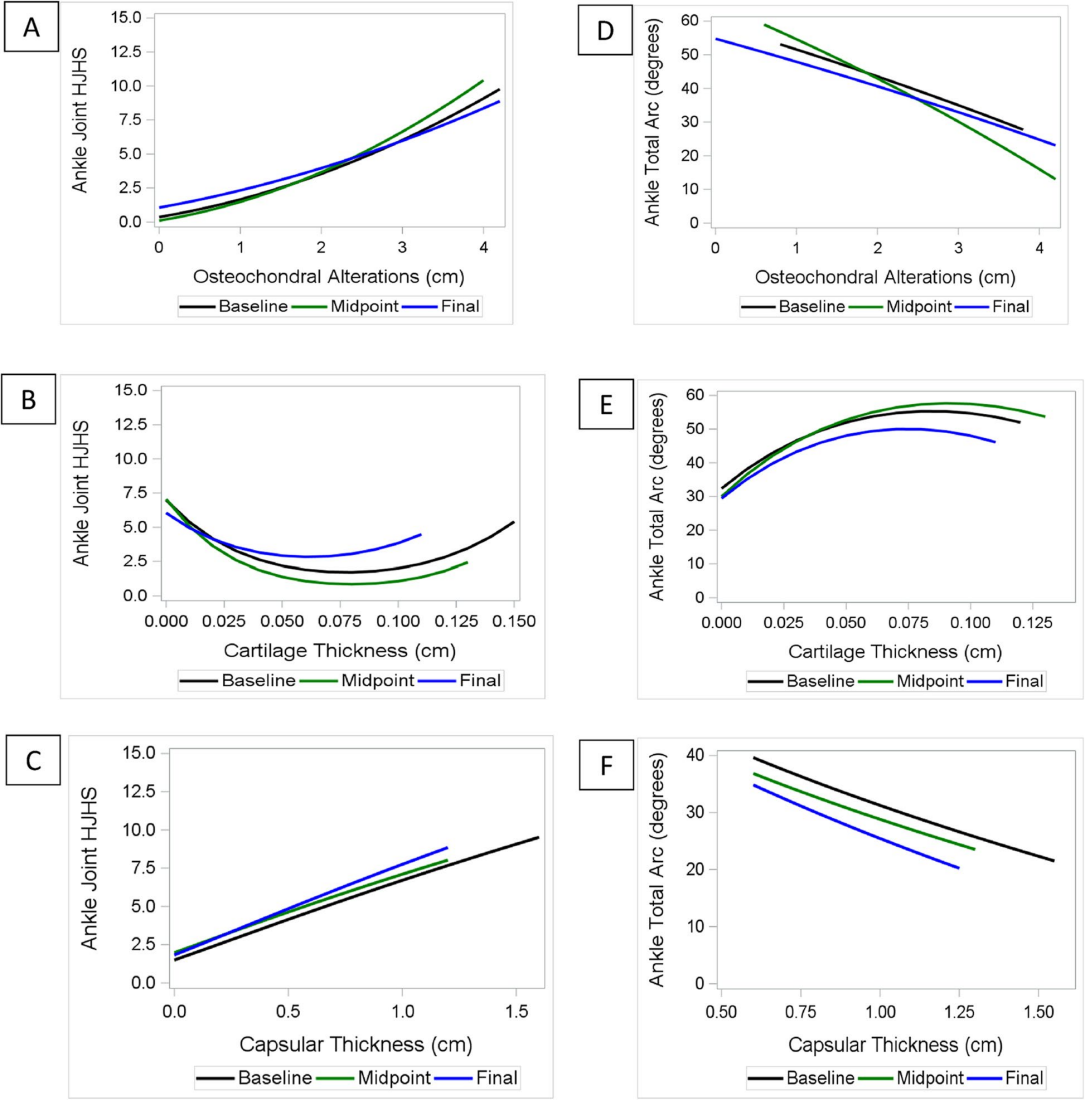
Ankle: Association of Joint _Tissue_Activity and Damage Exam variables with HJHS and total arc at each time point. Each patient had three time points (initial, midpoint, and final clinic visit). At each time point, the association is represented between HJHSs and JADE variables: **a** length of osteochondral alterations; **b** capsular thickness; and **c** cartilage thickness. At each time point, the association is represented between total arcs and JADE variables: **d** length of osteochondral alterations; **e** capsular thickness; and **f** cartilage thickness. The regression equation for each plot is shown in Table S4. JADE: Joint _Tissue_Activity and Damage Exam; HJHS: Hemophilia Joint Health Score

At each visit there was a positive association between HJHS with increasing length of osteochondral alterations on the talar dome and greater capsular extension in the anterior tibio-talar recess (Fig. 3a and 3c, Supplement 1: Table S6). Regarding the cartilage thickness on the talar dome, the HJHS was lowest (≈0–2) at a cartilage thickness of ≈0.05–0.1 cm, but increased when cartilage thickness was below 0.05 cm or above 0.1 cm (Fig. 3b; normal range reported between 0.1–0.17 mm [16]).

Changes in total arc followed a similar pattern. At each visit decreasing total arc was associated with increasing length of osteochondral alteration and greater capsular expansion (Fig. 3d and 3f, Supplement 1: Table S7). As for the HJHS, deteriorating total arc was observed when cartilage thickness was below 0.075 or above 0.1 cm (Fig. 3e).

### JADE version 2 protocol update

For elbow and ankle, the sentinel positions did not change from the prior version because the corresponding associations of osteochondral alterations, cartilage thickness, and soft tissue measurements with HJHS and total arc made at the three visits were consistent.

For the knee the sentinel position at the patellofemoral knee joint space in short axis remained because the corresponding associations of osteochondral and cartilage measurements with HJHS and total arc made at the three visits were consistent. However, assessments of the anterior and posterior condyles in long axis did not show any correlations, were therefore not deemed useful, and were removed from the JADE protocol. The corresponding associations of clinical and functional joint health with measurements of soft tissue expansion in the medial and lateral recesses were consistent and will therefore be continued. The 2^nd^ edition of the JADE protocol is attached as Supplement 2.

### Evaluation of acute painful joints

Nineteen patients experienced 33 painful episodes (ankle: *n* = 15, elbow: *n* = 9, knee: *n* = 7, shoulder: *n* = 1, hip: *n* = 1), of which 24 episodes (72%) were associated with bleeding, and 9 episodes were not (28%), as confirmed by MSKUS. MSKUS evaluation revealed that patient-perceived pain etiology, i.e. presence or absence of hemarthrosis, was correct in 20 instances (60%). MSKUS confirmed that 16/24 (66%) perceived bleeding episodes were indeed hemarthroses, while absence of hemarthrosis was confirmed for 4/9 (44%) perceived non-bleeding episodes.

## Discussion

In this cohort the associations between intra-articular structural measurements using the JADE protocol with clinical HJHSs and total arc in hemophilic joints (elbows, knees, and ankles) were reproduced at each visit. The cross-sectional assessments were consistent over a sufficiently long time (study period of averaging just over one and a half years) during which joint health changes are expected to occur in aging PWH [17]. Moreover, consistency was maintained despite a broad spectrum of hemophilic joint manifestations, evident by a wide range of HJHSs.

We have previously reported the JADE associations with a clinical parameter (HJHS), and a functional assessment (total arc), for hemophilia arthropathy at the baseline visit for this cohort [4]. The analysis reported here adds the consistency of these associations at three clinic visits within a 1- to 3-year period in the same cohort. Specifically, at each visit, deteriorating HJHS and total arc correlated with osteochondral alterations and soft tissue expansion measured by POC MSKUS in the expected direction. However, both elbow and knee showed a U-shaped curve for HJHS in relation to cartilage thickness. Total arc increased with cartilage thickness to reach a plateau for elbow and ankle, with the knee showing a decline with greater thickness. When the cartilage thickness was measured within or close to normal range, HJHSs scores were lowest and total arcs measurements were highest, e.g. joint health was at its best. Compared with healthy individuals, patients with osteoarthritis may have cartilage changes in both directions (thinning and thickening) as detected by Magnet Resonance Imaging [18]. Cartilage thickening or hypertrophy may occur prior to net cartilage loss observed in more advanced stages. In animal studies, increasing cartilage thickness may capture degenerative arthritis, including de novo synthesis of matrix content, hypertrophy of the cartilage and synovium, and/or fibrosis [19]. Here we observed not only cartilage thinning (as expected) but also cartilage thickening, both of which were associated with poorer joint health outcomes. We speculate that these changes may represent different stages of osteoarthritis in hemophilic joints, necessitating larger longitudinal studies to delineate associated qualitative structural changes such as loss of cartilage clarity and increase in echogenicity [20]. Based on these findings new studies are clearly needed to investigate ultrasonographic features of cartilage thickening, echogenic and structural changes in hemophilic joints in relation to joint health and function, including correlation with visual and histological examinations of live joint explants or cadavers.

The serial cross-sectional associations of direct tissue measurements by MSKUS with HJHS and/or total arc observed in this cohort are another step forward in the process of validating the JADE protocol in iterative fashion. An updated version 2 of the JADE protocol (2^nd^ edition, Supplement 2) was developed by removing several transducer positions for the knee, which were supposed to provide views depicting osteochondral and cartilage integrity on the medial and lateral anterior and posterior condyles. These positions were removed because of lack of associations with clinical and functional joint health parameters. In contrast, cartilage measurements in the femoro-patellar space proved to have associations with HJHS and total arc at each visit. Based on these adjustments, the new JADE 2 version views that are focused on direct tissue measurements include 3 elbow views, 3 knee views (previously 7) and 2 ankle views. The optional views to enable assessments of other musculoskeletal abnormalities in qualitative fashion, including (bloody) effusions, remained unchanged.

Image acquisition including measurements is quick, and takes only ≈1–2 min per view. Therefore, with the extraneous knee views removed, JADE Version 2 provides a slimmer, and more streamlined tool for ambulatory POC imaging. MSKUS, using the JADE protocol views assessing joint recesses, proved also to be a valuable tool to assess the occurrence of hemarthrosis during painful joint episodes. This appears clinically valuable based on the unreliability of patient-perceived symptoms related to hemarthrosis [21, 22], which has resulted in frequent use of MSKUS for this indication in hemophilia clinics [23].

The study limitations include: 1) lack of applicability to children, 2) inability of MSKUS to delineate deep joint structures, 3) unblinded design by the providers performing MSKUS to the HJHS and total arc, 4) incapability of POC MSKUS to provide a full diagnostic ultrasound evaluation due to restricted sentinel positions, and 5) no generalization to external populations. Also, the study assumes that the alterations of joint tissue structures are mostly related to the sequelae of hemophilic bleeding. Contributions of early onset osteoarthritis or crystal arthritis appear unlikely given the young age of the cohort (median age = 36 years). Similarly unlikely appears the presence of inflammatory/rheumatoid arthritis based on the absence of their clinical characteristics.

## Conclusions

This study demonstrated that serial cross-sectional associations between direct tissue measurements, made with POC MSKUS using the JADE protocol, and joint health outcomes were reproduced on three occasions separated by many months for 8 of the 12 sentinel transducer positions used for measurements in ankles, elbows, and knees. Joint health outcomes were not limited to clinical assessments by HJHS, but also included functional assessments (total arc) as an important parameter for day-to-day mobility. Tissues measured comprised soft tissue (synovial) expansion in the joint recesses as well as osteochondral alterations and cartilage thickness. Seeking to prove the value of transducer positions (views) through repetitive associations with clinical and functional parameters over time resulted in the elimination of 4 views from the JADE protocol, while strengthening the validity of the remaining 8 views. These 8 views (3 for elbow, 3 for knee, 2 for ankle) should provide a rapid POC tool for objective and clinically/functionally meaningful joint tissue measurements in clinical practice.

Therefore, these findings advance the JADE protocol validation on several levels, and enabled iterative adaptations resulting in the JADE protocol version 2, attached to this manuscript.

## Supplementary Information

**Additional file 1.**

**Additional file 2.**

## Data Availability

The datasets used and/or analyzed during this current study are available from the corresponding author on reasonable request.

## Abbreviations

MSKUS: Musculoskeletal ultrasound
POC: Point-of-care
HJHS: Hemophilia Joint Health Score
JADE: Joint _Tissue_Activity and Damage Exam
IQR: Interquartile
PWH: Person with hemophilia
OMERACT: Outcomes Measures in Rheumatology
ROM: Range of motion
