# Supplementary material for: Consistency of serial ultrasonographic joint tissue measurements by the Joint tissueActivity and Damage Exam (JADE) protocol in relation to hemophilic joint health parameters
Source: BMC Musculoskelet Disord. 2023 Apr 15;24:299. doi: 10.1186/s12891-023-06419-5 (PMC10105411; doi:10.1186/s12891-023-06419-5)
Supplement: Supplementary file 1 — Additional file 1. [file 12891_2023_6419_MOESM1_ESM.docx]

Supplement 1: Tables

**Consistency of Serial Ultrasonographic Joint Tissue Measurements by the Joint _tissue_Activity and Damage Exam (JADE) protocol in Relation to Hemophilic Joint Health Parameters**

Manuscript category: Original article

Richard F.W. Barnes Ph.D., M.P.H.^1^, Cris Hanacek D.O.^1^, Andres Flores M.S., A.T.C. ^1^, Peter Aguero P.T., D.P.T., R.M.S.K. ^2^, Bruno Steiner P.T., D.P.T., L.M.T., R.M.S.K. ^3^, Cindy Bailey P.T., D.P.T., O.C.S., S.C.S. ^4^, A.T.C., Doris Quon M.D. Ph.D. ^4^, Rebecca Kruse-Jarres M.D., M.P.H. ^3^, Annette von Drygalski, M.D.. Pharm.D., R.M.S.K. ^1^

^1^Department of Medicine, Division of Hematology/Oncology, University of California San Diego, San Diego, California, USA

^2^Department of Physical Medicine and Rehabilitation, University of California San Diego, San Diego, California, USA

^3^Washington Center for Bleeding Disorders, Seattle, WA, USA

^4^Orthopaedic Hemophilia Treatment Center at Orthopaedic Institute for Children, Los Angeles, California, CA, USA

Table S1a. The effect of transforming joint HJHS and Total arc values that were not normally distributed. The smaller the value of *D* the closer the fit to a normal distribution.

Hemophilia Joint Health Score

| **Joint** | **Distribution** | | | |
| --- | --- | --- | --- | --- |
|  | **HJHS** | | **√(0.5 + HJHS)** | |
|  | **D** | **p** | **D** | **p** |
| Elbow | 0.222 | <0.010 | 0.232 | <0.010 |
| Knee | 0.224 | <0.010 | 0.181 | <0.010 |
| Ankle | 0.135 | <0.010 | 0.145 | <0.010 |

Total Arc

| **Joint** | **Distribution** | | | |
| --- | --- | --- | --- | --- |
|  | **Total arc** | | ***Y* = *ln*(170 – Total arc)** | |
|  | **D** | **p** | **D** | **p** |
| Elbow | 0.178 | <0.010 | 0.134 | <0.010 |
| Knee | 0.208 | <0.010 | 0.119 | <0.010 |
| Ankle | 0.138 | <0.010 | 0.130 | <0.010 |

Table S1b. The effect of transforming joint HJHS and Total arc values on the distribution of model residuals. All models were the outcome on length of osteochondral alterations. The smaller the value of *D* the closer the fit to a normal distribution.

Hemophilia Joint Health Score

| **Joint** | **Distribution of residuals** | | | |
| --- | --- | --- | --- | --- |
|  | **HJHS** | | **√(0.5 + HJHS)** | |
|  | **D** | **p** | **D** | **p** |
| Elbow | 0.158 | <0.010 | 0.101 | 0.044 |
| Knee | 0.076 | >0.150 | 0.082 | >0.150 |
| Ankle | 0.081 | >0.150 | 0.062 | >0.150 |

Total Arc

| **Joint** | **Distribution of residuals** | | | |
| --- | --- | --- | --- | --- |
|  | **Total arc** | | ***Y* = *ln*(170 – Total arc)** | |
|  | **D** | **p** | **D** | **p** |
| Elbow | 0.143 | <0.010 | 0.136 | <0.010 |
| Knee | 0.115 | 0.035 | 0.089 | >0.150 |
| Ankle | 0.067 | >0.150 | 0.079 | >0.150 |

Table S1c. The effect of transforming joint HJHS and Total arc values on the distribution of model residuals. All models were the outcome on length of osteochondral alterations. The larger the value of *t* the more precise the estimate.

Hemophilia Joint Health Score

| **Joint** | **Untransformed**  **(Hjhs_total_score_hjhs)** | | | | **Transformed**  (**√(0.5 + Hjhs_total_score_hjhs)** | | | |
| --- | --- | --- | --- | --- | --- | --- | --- | --- |
|  | **Estimate** | **SE** | **t** | **p** | **Estimate** | **SE** | **t** | **p** |
| Elbow | 1.2141 | 0.3740 | 3.25 | 0.0025 | 0.3619 | 0.09290 | 3.90 | 0.0004 |
| Knee | 2.3373 | 0.3594 | 6.50 | <0.0001 | 0.5751 | 0.08572 | 6.71 | <0.0001 |
| Ankle | 2.3142 | 0.4715 | 4.91 | <0.0001 | 0.5422 | 0.1041 | 5.21 | <0.0001 |

Total Arc

| **Joint** | **Untransformed**  **(Total arc)** | | | | **Transformed**  ***Y* = *ln*(170 – Total arc)** | | | |
| --- | --- | --- | --- | --- | --- | --- | --- | --- |
|  | **Estimate** | **SE** | **t** | **p** | **Estimate** | **SE** | **t** | **p** |
| Elbow | -9.6653 | 2.1225 | -4.55 | <0.0001 | 0.2233 | 0.04165 | 5.36 | <0.0001 |
| Knee | -9.2346 | 2.2771 | -4.06 | 0.0004 | 0.1753 | 0.04230 | 4.15 | 0.0003 |
| Ankle | -8.8338 | 2.1207 | -4.17 | 0.0002 | 0.06794 | 0.01675 | 4.06 | 0.0002 |

Table S2: Curves for each visit describing Elbow joint HJHS in relation to each JADE measure. The outcome is *√*(0.5 + HJHS). The tests for coinciding curves examine the null hypothesis that the curves are parallel and their intercepts are equal**.** They compare the curves for Midpoint versus Baseline and the curves for Final versus Baseline.

| **Visit** | **Independent variable, X** | **Regression equation for curve** | | | **Tests for coinciding curves** | | |
| --- | --- | --- | --- | --- | --- | --- | --- |
|  |  | **Intercept** | **Regression coefficient, b_1_ for X_1_** | **Regression coefficient b_2_ for X_2_** | **Test for parallelism (visit*X_1_)**  **P-value** | **Test for parallelism (visit*X_2_)**  **P-value** | **Test for equal intercepts: P-value** |
| Baseline | Length of osteochondral alteration | 0.50  (-0.17, 1.18) | 0.36  (0.17, 0.55) |  |  |  |  |
| Midpoint | Length of osteochondral alteration | -0.23  (-0.71, 0.26) | 0.60  (0.47, 0.74) |  | 0.19 |  | 0.27 |
| Final | Length of osteochondral alteration | 0.06  (-0.42, 0.53) | 0.52  (0.39, 0.66) |  | 0.40 |  | 0.55 |
| Baseline | Cartilage thickness | 1.46  (1.23, 1.70) | -16.66  (-19.81, -13.52) | 131.23  (53.20, 209.26) |  |  |  |
| Midpoint | Cartilage thickness | 1.30  (1.09, 1.51) | -12.91  (-15.64, -10.18) | 143.81  (100.77, 186.84) | 0.03 | 0.99 | 0.24 |
| Final | Cartilage thickness | 1.42  (1.17, 1.67) | -11.76  (-14.95, -8.56) | 104.87  (64.35, 145.40) | 0.02 | 0.64 | 0.37 |
| Baseline | Ln(Fat pad area) | -1.68  (-2.56, -0.80) | 3.02  (2.27, 3.77) |  |  |  |  |
| Midpoint | Ln(Fat pad area) | -0.62  (-1.39, 0.14) | 2.12  (1.47, 2.77) |  | 0.04 | 0.06 |  |
| Final | Ln(Fat pad area) | -1.75  (-2.62, -0.89) | 3.16  (2.41, 3.91) |  | 0.68 | 0.80 |  |

Table S3: Curves for each visit describing Elbow Total arc in relation to each JADE measure. The outcome is *ln*(170 – Total arc). The tests for coinciding curves examine the null hypothesis that the curves are parallel and their intercepts are equal**.** They compare the curves for Midpoint versus Baseline and the curves for Final versus Baseline.

| **Visit** | **Independent variable, X** | **Regression equation for curve** | | | **Tests for coinciding curves** | | |
| --- | --- | --- | --- | --- | --- | --- | --- |
|  |  | **Intercept** | **Regression coefficient, b_1_ for X_1_** | **Regression coefficient b_2_ for X_2_** | **Test for parallelism (visit*X_1_)**  **P-value** | **Test for parallelism (visit*X_2_)**  **P-value** | **Test for equal intercepts: P-value** |
| Baseline | Length of osteochondral alteration | 2.99  (2.69, 3.29) | 0.22  (0.14, 0.31) |  |  |  |  |
| Midpoint | Length of osteochondral alteration | 2.79  (2.56, 3.02) | 0.30  (0.23, 0.36) |  | 0.16 |  | 0.27 |
| Final | Length of osteochondral alteration | 2.83  (0.61, 3.06) | 0.27  (0.21, 0.34) |  | 0.82 |  | 0.82 |
| Baseline | Cartilage thickness | 3.56  (3.43, 3.68) | -7.50  (-9.09, 3.68) | 47.37  (13.99, 80.76) |  |  |  |
| Midpoint | Cartilage thickness | 3.49  (3.37, 3.60) | -5.58  (-6.91, -4.25) | 64.25  (42.53, 85.96) | 0.15 | 0.42 | 0.32 |
| Final | Cartilage thickness | 3.58  (3.46, 3.71) | -6.33  (24.47, 57.95) | 41.21  (24.47, 57.95) | 0.62 | 0.96 | 0.97 |
| Baseline | Ln(Fat pad area) | 2.35  (1.90, 2.80) | 1.23  (0.85, 1.61) |  |  |  |  |
| Midpoint | Ln(Fat pad area) | 2.66  (2.24, 3.08) | 0.97  (0.62, 1.32) |  | 0.76 |  | 0.73 |
| Final | Ln(Fat pad area) | 2.11  (1.63, 2.58) | 1.46  (1.05, 1.87) |  | 0.23 |  | 0.26 |

Table S4: Curves for each visit describing Knee joint HJHS in relation to each JADE measure. The outcome is *√*(0.5 + HJHS). The tests for coinciding curves examine the null hypothesis that the curves are parallel and their intercepts are equal**.** They compare the curves for Midpoint versus Baseline and the curves for Final versus Baseline.

| **Visit** | **Independent variable, X** | **Regression equation for curve** | | | **Tests for coinciding curves** | | |
| --- | --- | --- | --- | --- | --- | --- | --- |
|  |  | **Intercept** | **Regression coefficient, b_1_ for X_1_** | **Regression coefficient b_2_ for X_2_** | **Test for parallelism (visit*X_1_)**  **P-value** | **Test for parallelism (visit*X_2_)**  **P-value** | **Test for equal intercepts: P-value** |
| Baseline | Length of osteochondral alteration | 1.42  (1.15, 1.67) | 0.59  (0.41, 0.77) | 0.08  (-0.05, 0.21) |  |  |  |
| Midpoint | Length of osteochondral alteration | 1.61  (1.31, 1.91 | 0.28  (0.09, 0.47) | 0.02  (-0.12, 0.17) | 0.05 | 0.97 | 0.53 |
| Final | Length of osteochondral alteration | 1.47  (1.23, 1.72) | 0.38  (0.22, 0.53) | 0.11  (0.01, 0.21) | 0.02 | 0.87 | 0.49 |
| Baseline | Cartilage thickness | 3.20  (2.70, 3.70) | -6.45  (-8.29, -4.62) |  |  |  |  |
| Midpoint | Cartilage thickness | 2.80  (2.32, 3.28) | -4.76  (-6.46, -3.05) |  | 0.44 | 0.44 |  |
| Final | Cartilage thickness | 2.74  (2.17, 3.31) | -4.70  (-6.83, -2.57) |  | 0.13 | 0.12 |  |
| Midpoint | Ln(Soft tissue expansion) | 1.99  (1.72, 2.27) | 5.07  (2.97, 7.17) | -4.47  (-23.98, 15.04) | 0.93 | 0.43 | 0.25 |
| Final | Ln(Soft tissue expansion) | 2.07  (1.83, 2.31) | 6.59  (4.83, 8.36) | -6.55  (-23.15, 10.05) | 0.53 | 0.97 | 0.28 |

Table S5: Curves for each visit describing Knee Total arc in relation to each JADE measure. The outcome is *ln*(170 – Total arc). The tests for coinciding curves examine the null hypothesis that the curves are parallel and their intercepts are equal**.** They compare the curves for Midpoint versus Baseline and the curves for Final versus Baseline.

| **Visit** | **Independent variable, X** | **Regression equation for curve** | | | **Tests for coinciding curves** | | |
| --- | --- | --- | --- | --- | --- | --- | --- |
|  |  | **Intercept** | **Regression coefficient, b_1_ for X_1_** | **Regression coefficient b_2_ for X_2_** | **Test for parallelism (visit*X_1_)**  **P-value** | **Test for parallelism (visit*X_2_)**  **P-value** | **Test for equal intercepts: P-value** |
| Baseline | Length of osteochondral alteration | 3.08  (2.76, 3.40) | 0.17  (0.09, 0.26) |  |  |  |  |
| Midpoint | Length of osteochondral alteration | 3.15  (2.91, 3.40) | 0.13  (0.06, 0.19) |  | 0.91 |  | 0.71 |
| Final | Length of osteochondral alteration | 3.12  (2.81, 3.44) | 0.14  (0.05, 0.23) |  | 0.29 |  | 0.61 |
|  |  |  |  |  |  |  |  |
| Baseline | Cartilage thickness | 4.46  (4.25, 4.68) | -6.07  (-7.64, -4.51) | 10.40  (6.56, 12.25) |  |  |  |
| Midpoint | Cartilage thickness | 4.35  (4.10, 4.60) | -6.52  (-8.24, -4.80) | 12.14  (8.70, 15.58) | 0.31 | 0.07 | 0.95 |
| Final | Cartilage thickness | 4.38  (3.80, 4.95) | -7.26  (-11.51, -3.01) | 13.11  (5.45, 20.77) | 0.08 | 0.07 | 0.03 |
| Baseline | Ln(Soft tissue expansion) | 3.26  (2.94, 3.57) | 1.76  (0.82, 2.71) |  |  |  |  |
| Midpoint | Ln(Soft tissue expansion) | 3.35  (3.00, 3.71) | 1.37  (0.25, 2.48) |  | 0.28 |  | 0.23 |
| Final * | Ln(Soft tissue expansion) | 3.91  (3.76, 4.07) | 4.14  (2.98, 5.31) | -11.30  (-18.75, -3.86) |  |  |  |

While the Baseline and Midpoint curves coincide, the Final curve differs from both.Table S6: Curves for each visit describing Ankle joint HJHS in relation to each JADE measure. The outcome is *√*(0.5 + HJHS). The tests for coinciding curves examine the null hypothesis that the curves are parallel and their intercepts are equal**.** They compare the curves for Midpoint versus Baseline and the curves for Final versus Baseline.

| **Visit** | **Independent variable, X** | **Regression equation for curve** | | | **Tests for coinciding curves** | | |
| --- | --- | --- | --- | --- | --- | --- | --- |
|  |  | **Intercept** | **Regression coefficient, b_1_ for X_1_** | **Regression coefficient b_2_ for X_2_** | **Test for parallelism (visit*X_1_)**  **P-value** | **Test for parallelism (visit*X_2_)**  **P-value** | **Test for equal intercepts: P-value** |
| Baseline | Length of osteochondral alteration | 0.93  (0.36 1.50) | 0.54  (0.33, 0.75) |  |  |  |  |
| Midpoint | Length of osteochondral alteration | 0.77  (0.15, 1.40) | 0.63  (0.40, 0.70) |  | 0.29 |  | 0.20 |
| Final | Length of osteochondral alteration | 1.25  (0.62, 1.87) | 0.43  (0.22, 0.65) |  | 0.22 |  | 0.28 |
| Baseline | Ln(Cartilage thickness) | 1.95  (1.62, 2.29) | -20.45  (-28.64, -12.25) | 223.32  (51.64, 395.0) |  |  |  |
| Midpoint | Ln(Cartilage thickness) | 1.78  (1.37, 2.18) | -25.75  (-35.06, -16.43) | 269.19  (7.50, 530.9) | 0.70 | 0.97 | 0.73 |
| Final | Ln(Cartilage thickness) | 2.02  (1.63, 2.40) | -12.65  (-21.72, -3.57) | 204.95  (-45.14, 455.1) | 0.53 | 0.71 | 0.99 |
| Baseline | Ln(Capsular thickness) | 1.41  (0.77, 2.06) | 1.83  (0.60, 3.07) |  |  |  |  |
| Midpoint | Ln(Capsular thickness) | 1.57  (1.00, 2.14) | 1.71  (0.57, 2.85) |  | 0.06 |  | 0.06 |
| Final | Ln(Capsular thickness) | 1.52  (0.91, 2.04) | 1.94  (0.71, 3.18) |  | 0.63 |  | 0.39 |

Table S7: Curves for each visit describing Ankle Total arc in relation to each JADE measure. The outcome is *ln*(170 – Total arc). The outcome is √(0.5 + HJHS). The tests for coinciding curves examine the null hypothesis that the curves are parallel and their intercepts are equal**.** They compare the curves for Midpoint versus Baseline and the curves for Final versus Baseline.

| **Visit** | **Independent variable, X** | **Regression equation for curve** | | | **Tests for coinciding curves** | | |
| --- | --- | --- | --- | --- | --- | --- | --- |
|  |  | **Intercept** | **Regression coefficient, b_1_ for X_1_** | **Regression coefficient b_2_ for X_2_** | **Test for parallelism (visit*X_1_)**  **P-value** | **Test for parallelism (visit*X_2_)**  **P-value** | **Test for equal intercepts: P-value** |
| Baseline | Length of osteochondral alteration | 4.71  (4.61, 4.81) | 0.07  (0.03, 0.10) |  |  |  |  |
| Midpoint | Length of osteochondral alteration | 4.65  (4.56, 4.75) | 0.10  (0.06, 0.13) |  | 0.41 |  | 0.69 |
| Final | Length of osteochondral alteration | 4.75  (4.65, 4.84) | 0.06  (0.02, 0.09) |  | 0.98 |  | 0.66 |
| Baseline | Ln(Cartilage thickness) | 4.82  (4.77, 4.87) | -2.90  (-4.13, -1.67) | 27.83  (2.65, 53.02) |  |  |  |
| Midpoint | Ln(Cartilage thickness) | 4.82  (4.77, 4.87) | -3.37  (-4.68, -2.07) | 28.88  (2.08, 55.68) | 0.16 | 0.12 | 0.36 |
| Final | Ln(Cartilage thickness) | 4.83  (4.78, 4.89) | -2.63  (-3.96, -1.30) | 30.63  (-6.87, 68.12) | 0.22 | 0.480.36 | 0.68 |
| Baseline | Ln(Capsular thickness) | 4.74  (4.63, 4.85) | 0.28  (0.07, 0.48) |  |  |  |  |
| Midpoint | Ln(Capsular thickness) | 4.77  (4.68, 4.86) | 0.27  (0.08, 0.46) |  | 0.65 |  | 0.33 |
| Final | Ln(Capsular thickness) | 4.76  (4.67, 4.86) | 0.30  (0.11, 0.49) |  | 0.93 |  | 0.46 |
