# Supplementary material for: Consistency of serial ultrasonographic joint tissue measurements by the Joint tissueActivity and Damage Exam (JADE) protocol in relation to hemophilic joint health parameters
Source: BMC Musculoskelet Disord. 2023 Apr 15;24:299. doi: 10.1186/s12891-023-06419-5 (PMC10105411; doi:10.1186/s12891-023-06419-5)
Supplement: Supplementary file 2 — Additional file 2. [file 12891_2023_6419_MOESM2_ESM.docx]

Supplement 2: JADE protocol

Ultrasonography and Hemophilia –

J.A.D.E. Protocol – 2nd Edition


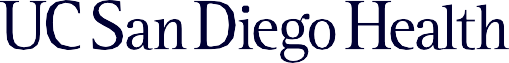


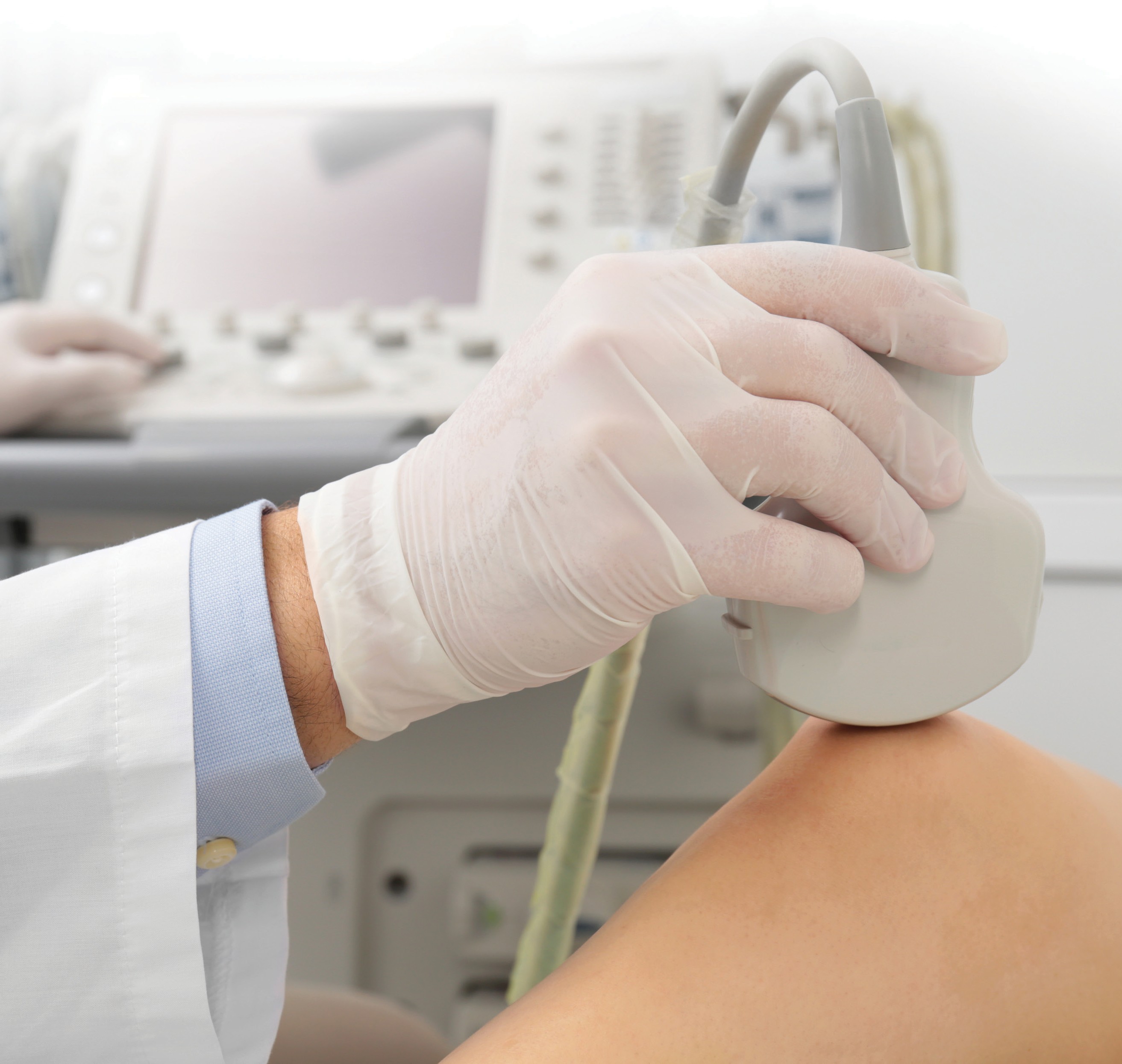


Ultrasonography and Hemophilia –

J.A.D.E. Protocol – 2nd Edition

Annette von Drygalski, MD, PharmD, RMSK Randy Moore, DC, RDMS, RMSK

Lena Volland, PT, DPT, OCS Eric Chang, MD

Peter Aguero, PT, DPT, RMSK

Table of Contents


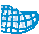

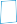

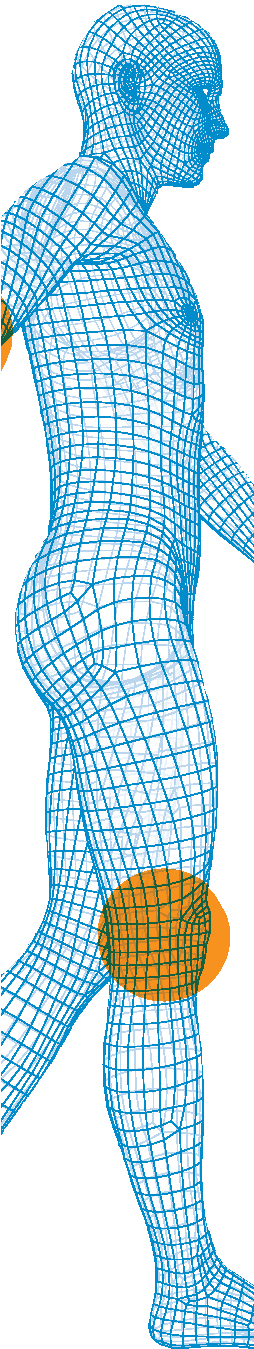

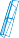

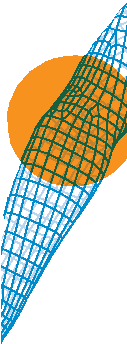

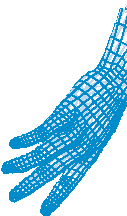

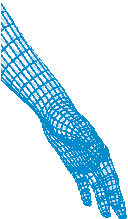

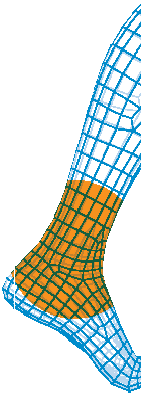

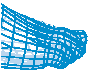


[Musculoskeletal Ultrasound 1](#_TOC_250007)

[Power Doppler Signal 3](#_TOC_250006)

[Detection of Effusions 4](#_TOC_250005)

[Elbow 5](#_TOC_250004)

[Knee 10](#_TOC_250003)

[Ankle 19](#_TOC_250002)

[About the Authors 26](#_TOC_250001)

[References 29](#_TOC_250000)

This protocol is copyrighted by The Regents of the University of California. The document and supporting materials are supplied “as is”, without any accompanying services from The Regents. The Regents does not warrant that the operation of the protocol will be uninterrupted or error-free. The end-user understands that the protocol was developed for program evaluation and assessment purposes and is advised not to rely exclusively on the protocol for any reason. In no event shall the University of California be liable to any party for direct, indirect, special, incidental, or consequential damages, including lost profits, arising out of the use of this protocol, even if the University of California has been advised of the possibility of such damage. The University of California specifically disclaims any warranties, including, but not limited to, the implied warranties of merchantability and fitness for a particular purpose. The protocol provided hereunder is on an “as is” basis, and the University of California has no obligations to provide maintenance, support, updates, enhancements, or modifications.

# Musculoskeletal Ultrasound

Patients with hemophilia often experience repetitive joint bleeding episodes leading to hemophilic arthropathy, which is most commonly seen in the elbow, knee, and ankle. There is a need to detect formation and progression of hemophilic arthropathy in a fast and convenient fashion to afford targeted management of joint abnormalities.

Traditionally, radiographic (Pettersson score), magnet resonance imaging (MRI; International Prophylaxis Study Group Score) and clinical (Hemophilia Joint Health Score) scoring algorithms are used to monitor joint health. However, radiographs are insensitive to soft tissue changes, MRIs are lengthy, often requiring contrast or sedation, and cannot be performed repeatedly for all joints, and, clinical scoring does not provide information on a tissue level. Musculoskeletal ultrasound (MSKUS) has emerged as a rapid, point- of-care (POC) imaging modality to detect bleeding episodes and to follow joint health longitudinally.

MSKUS is highly sensitive to soft tissue and osteochondral changes and can be performed frequently.

The Joint Tissue Activity and Damage Exam (J.A.D.E.) is a quantitative and concise MSKUS protocol evaluating soft tissue proliferation (joint activity) and alterations to bone and cartilage (joint damage) in hemophilic joints.

The principle of J.A.D.E. is inherent to precise measurements of intraarticular soft tissue expansion, cartilage thickness and osteochondral interface irregularities in elbows, knees, and ankles. Furthermore, the J.A.D.E. protocol employs sonopalpation to differentiate between effusions and soft tissue and permits charting of descriptive findings. Additionally, the J.A.D.E. protocol captures abnormal soft tissue microcirculation and vascularity changes by Power Doppler (PD) signals, altogether providing a versatile tool to dynamically follow the overall joint health status.

## J.A.D.E. Protocol

The J.A.D.E. protocol has been validated by OMERACT guidelines (omeract.org), and is continuously improved in an iterative process. This booklet provides the latest version (March 2022) with detailed descriptions of standardized transducer positions for image acquisition, as well as an atlas outlining sonoantomy and sonopathology with comprehensive explanations and examples of quantitative and qualitative J.A.D.E. assessments for the elbow, knee, and ankle.

The J.A.D.E. protocol provides obligatory views for rapid measurements to capture soft tissue and osteochondral changes, bleed detection views where effusions can be appreciated, as well as optional views useful for point-of-care (POC) assessments of joint abnormalities.

Standardized assessment sheets for clinical charting and research use can be downloaded from

go.ucsd.edu/3CEXO95. Equipment

Ultrasound machines can be leased or purchased from several companies and may be stationary or portable. The equipment should include a musculoskeletal setting, B mode (gray scale), a broad spectrum linear transducer (at least 8 MHZ), measurement tools, and PD function. Additional software capabilities,

such as tissue harmonic, spatial compounding, virtual convex and extended field of view (panoramic) imaging may be useful to optimize MSKUS. However, ultrasound technology is evolving rapidly, with handheld devices gaining traction. Transducers can be attached to cell phones or tablets, and the systems appear easy to navigate. Some brands offer tele-guidance features as well.

General Procedure Recommendations

During the scanning procedure the transducer orientation should be perpendicular to the bony landmark to visualize a bright, sharp, and hyperechoic cortical margin, which will serve as the basis of correct image acquisition. To improve visualization of structures the transducer can by dynamically translated, toggled (side-to-side angulation) or moved in a heel-toe fashion (front-back angulation). Continuous transducer position adjustment to keep the beam angle perpendicular will also limit artifacts, such as anisotropy that may result in misinterpretation of findings based on echogenicity. Dynamic joint motion can also be useful to examine the integrity of structures, such as ligaments and tendons, or fluid movements.

Soft Tissue Expansion Measurement

During all soft tissue (content) expansion measurements, adequate compression with the probe is necessary to displace any fluid in the recess. This compression will provide a more accurate measurement of soft tissue.

Examiner Qualifications

The J.A.D.E. protocol can be utilized by health care professionals, such as physicians, physical therapists, nurse practitioners, physician assistants, and nurses, trained in MSKUS. We recommend MSKUS

“hands-on” training through an accredited program, providing general MSKUS knowledge encompassing ultrasound physics, anatomy, sonoantomy and sonopathology, as well as MSKUS knowledge specific

to hemophilic arthropathy. We recommend frequent use of MSKUS to sharpen operator expertise and continuous education to maintain and improve skills.

Several courses are being provided by the UC San Diego Health Hemophilia and Thrombosis Treatment Center, including:

- Musculoskeletal Ultrasound Training for Arthritic Conditions: Ankle, Knee and Elbow – Basics and Techniques Online Program (CME accredited)
- Musculoskeletal Ultrasound Training in Hemophilia Online Program (CME accredited)
- Teleguided Hands-on Training for Ankle, Knee and Elbow (not CME accredited)
- Musculoskeletal Ultrasound Training for Hemophilia and Other Arthritic Conditions Live Course (CME accredited)
- Biweekly international grand rounds

For more information, please visit go.ucsd.edu/3CEXO95. Time Requirements

The J.A.D.E. protocol can be utilized in its entirety with an estimated time requirement of approximately 10 minutes per joint for the obligatory transducer positions required for measurements and/or detection of hemarthrosis. Certain elements of the J.A.D.E. protocol are optional, and can be added for POC questions, such as assessment of a specific abnormality (for instance tendon sprain) with a time requirement of a few minutes only.

# Power Doppler Signal

The presence of intraarticular soft tissue PD signals is abnormal and indicates inflammatory microvascular changes. Abnormal vessels in hemophilic joints can be leaky and fragile. Thus, they can easily contribute to repetitive or perpetuated joint bleeding. Consequently, it is important to incorporate PD signal analysis to evaluate joint health status.

PD signals are assessed in each of the standardized transducer positions and scored semi-quantitatively (0=no signal; 1=small spots; 2=confluent vessels in <50% tissue of interest; 3=confluent vessels in ≥ 50% tissue of interest).

## Power Doppler Score

Depicted are scoring examples in specific joint locations. The asterisk (*) indicates effusion. Note that the PD signal acquisition rectangle is often larger than the tissue of interest, which may be a smaller area within the rectangle.

1 2 3

Elbow


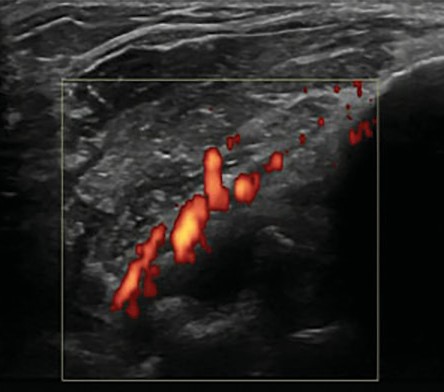

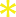

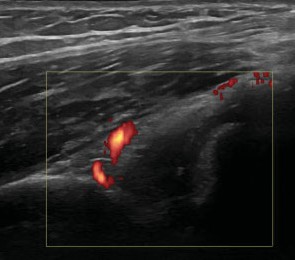

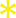

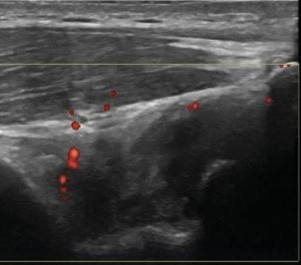

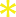


Olecranon fossa


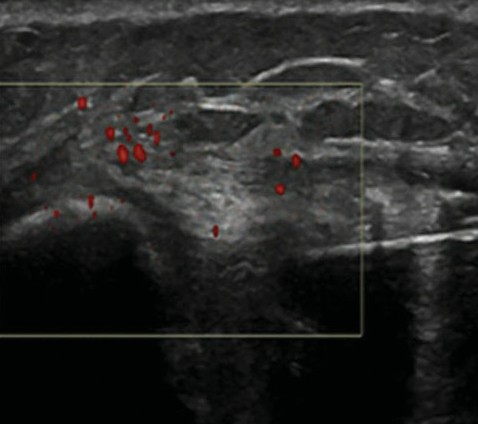

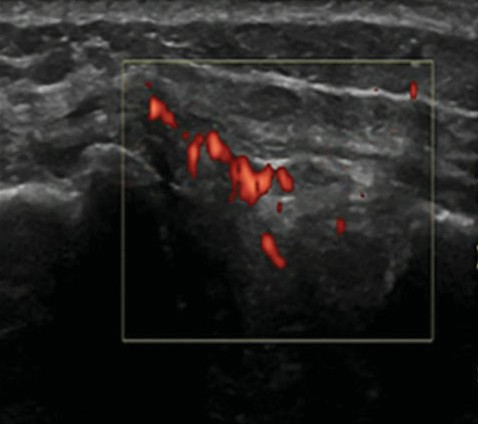
Knee


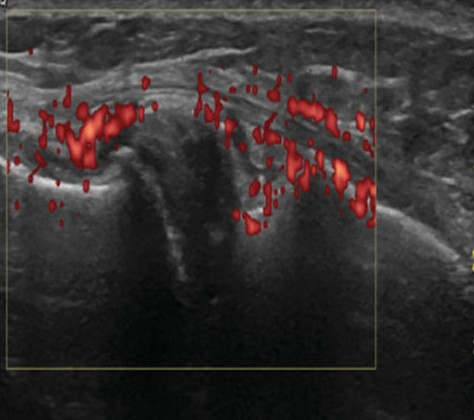

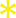


Medial meniscus


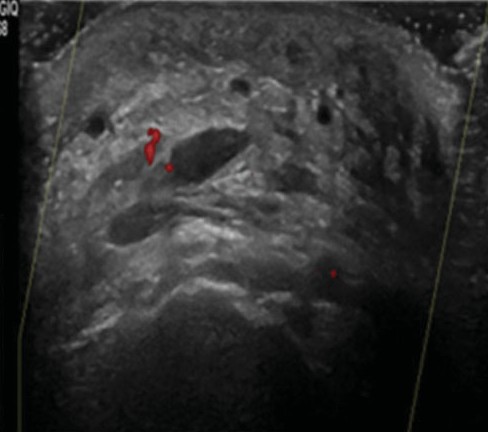
Ankle


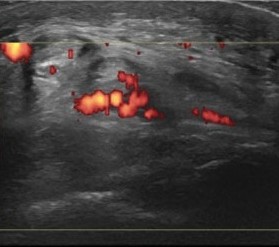

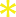

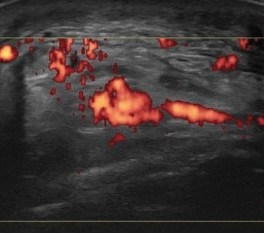

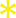


Tibiotalar joint

# Detection of Effusions

Joint spaces and recesses can be filled with fluid (serous or bloody) and/or expanding soft tissue, such as synovial hypertrophy, blood clots, lipoma arborescence, or fatty tissues. These components cannot be correctly identified by echotexture alone. Sonopalpation will differentiate between compressible fluid and non-compressible soft tissue and can be performed in each transducer position throughout the J.A.D.E. protocol. In contrast to soft tissue, fluid is compressible, moveable with dynamic motions, and avascular. Fluid will not exhibit PD signals. Effusion can be simple or complex. Simple effusions are anechoic, whereas complex effusions are hypoechoic, granular, with displaceable speckles or material. Simple effusions are usually serous, whereas complex effusions are considered to be bloody in the context of hemophilia. Blood clot formation will result in loss of or much decreased compressibility. Echogenicity patterns of blood clots are currently unknown.

Anechoic simple effusion (non bloody) in the suprapatellar bursa showing a black appearance and being fully compressible


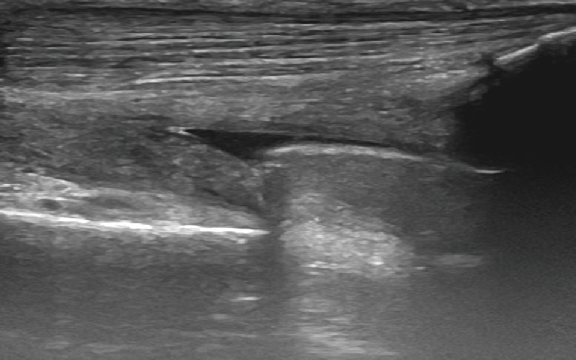

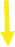


**Suprapatellar bursa**

**patella**

**femur**

Hypoechoic complex effusion (bloody) within the suprapatellar bursa displaying speckles and being compressible


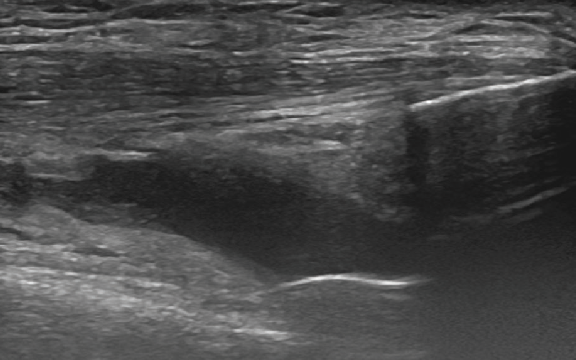

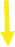


**Suprapatellar bursa**

**patella**

**femur**

Soft tissue expansion into the suprapatellar bursa appearing echogenic, but being noncompressible


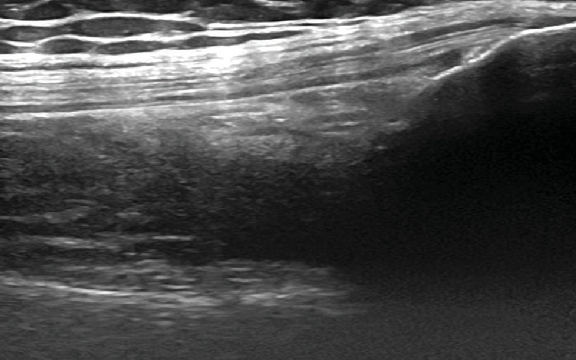

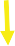


**patella**

**Suprapatellar bursa**

**femur**


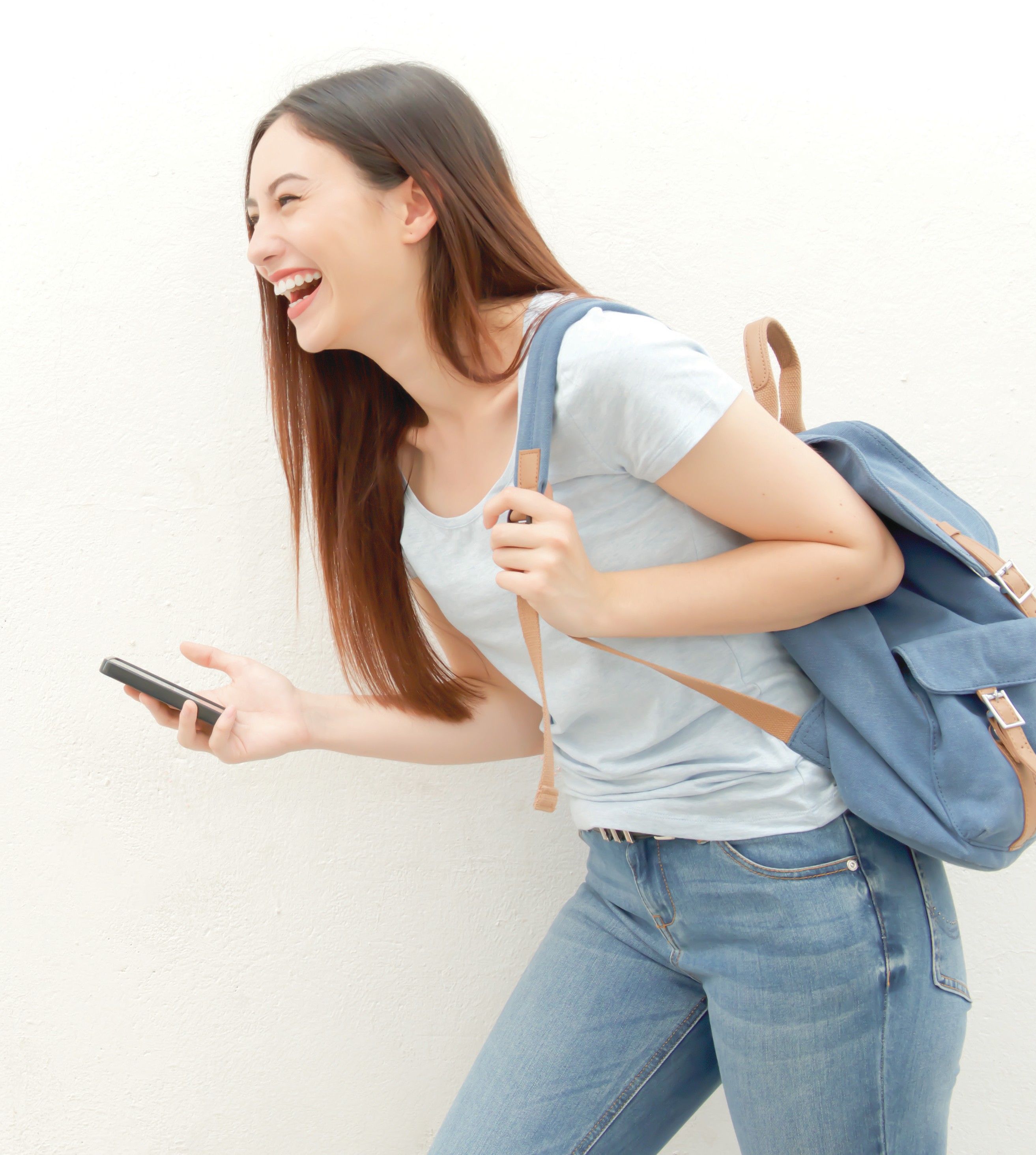


# Elbow

Ultrasonography and Hemophilia | J.A.D.E. Protocol | UC San Diego Health 5

## Anatomy of the Elbow

Joints

- Humeroulnar: Synovial hinge joint between humerus and ulnar
- Humeroradial: Synovial hinge joint between humerus and radius
- Proximal radioulnar: Synovial pivot joint between radius and ulna

Articulating Surfaces

- Humeroulnar joint: Trochlea of humeral condyle and trochlear notch of ulna
- Humeroradial joint: Capitulum of humeral condyle and head of the radius
- Proximal radioulnar: Head of the radius and radial notch of ulnar

Ligaments

- Lateral: Radial collateral ligament, annular ligament wrapping radial head
- Medial: Ulnar collateral ligament

Joint Motion

- Flexion and extension occurs in the humeroradial and humeroulnar joint
- Pronation and supination occurs in the radialulnar joint

Associated Muscles

- Flexion: Biceps brachii, brachialis, brachiradialis, pronator teres
- Extension: Triceps barchii, anconeus
- Pronation: Pronator teres, pronator quadratus
- Supination: Supinator, biceps brachii

Associated Nerves

- Anterior: Median, musculocutaneous, nerve
- Posterior: Ulnar nerve
- Lateral: Lateral cutaneous, posterior cutaneous, radial nerve
- Medial: Medial cutaneous nerve

Blood Supply

- Cubital anastomosis via branches of the brachial and deep brachial arteries; brachial artery splits into ulnar and radial artery slightly distal to

the elbow

## J.A.D.E. Views

Three views are provided for optimal assessment of soft tissue proliferation and inflammation, effusion detection, and osteochondral evaluation. These are focused on the antecubital fossa allowing assessment of the humeroradial and humeroulnar joint as well as the olecranon recess in the posterior elbow. It is recommended to complete all three transducer positions as a baseline exam for longitudinal follow-up, and detection of effusions. Fluid can accumulate in the anterior and posterior elbow region depending on the elbow position.

| BLEED DETECTION | |  |
| --- | --- | --- |
|  | Anterior elbow SAX Anterior elbow LAX Posterior elbow LAX |  |
|  | OBLIGATORY | |

OPTIONAL

None


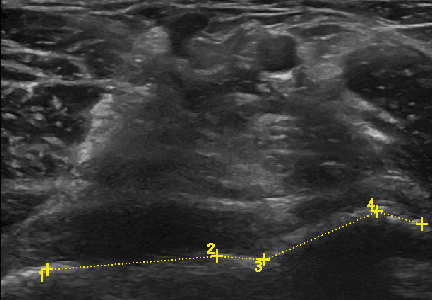


## Anterior Elbow SAX

Measurement of Osteochondral Interface Irregularities and Effusion/Bleed Detection Patient position: Supine, palm up with bolster under the hand to limit elbow extension

Axis: SAX

Probe position: In antecubital fossa

Bony landmarks: Ulnar trochlea (medial) and humeral capitulum (lateral)

Muscles: Brachialis (central), brachioradialis (lateral), pronator (medial), biceps brachialis tendon (superficial to brachialis)

Blood vessels: Brachial artery and vein

Nerves: Radial nerve (lateral), median nerve (medial)

Measurement: Individually measure any variation from hyperechoic, smooth, intact bony interface. Then add the values together for the total length of alterations.


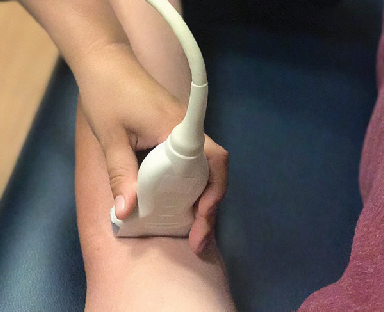
*Example*


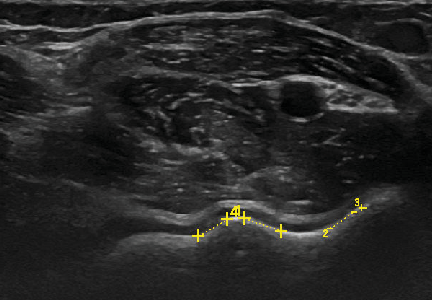


4 L 0.37 cm

3 L 0.02 cm

Total length: 0.39 cm


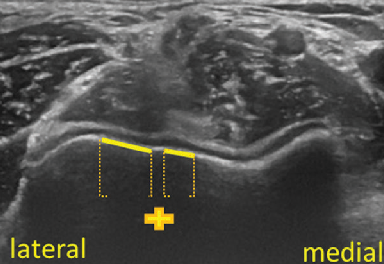

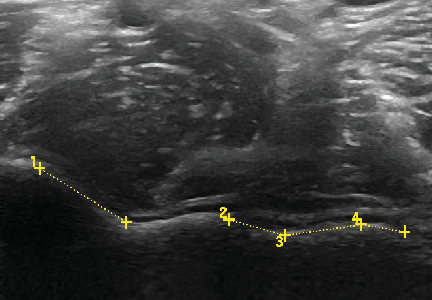
*Example*

| 1 L 0.99 cm |
| --- |
| 2 L 0.56 cm |
| 3 L 0.75 cm |
| 4 L 0.43 cm |

Total length: 2.73 cm

*Example*

| 1 L 1.93 cm |
| --- |
| 2 L 0.53 cm |
| 3 L 1.38 cm |
| 4 L 0.53 cm |

Total length: 4.37 cm

## Anterior Elbow LAX

Measurement of Cartilage Thickness and Effusion/Bleed Detection

Patient position: Supine, palm up with bolster under the hand to limit elbow extension

Axis: LAX

Probe position: Lateral aspect of the antecubital fossa over the humeroradial joint space

Bony landmarks: Humeral capitulum (proximal) and radial head (distal)

Muscles: Brachioradialis (superficial to humeroradial joint)

Blood vessels: None

Nerves: None

Measurement: Assess cartilage thickness at the apex and 0.25 cm distally. Assure that measurement is perpendicular to the bony margin.


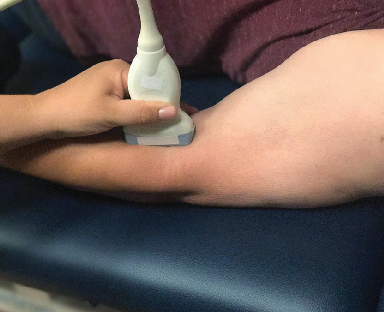
*Example*


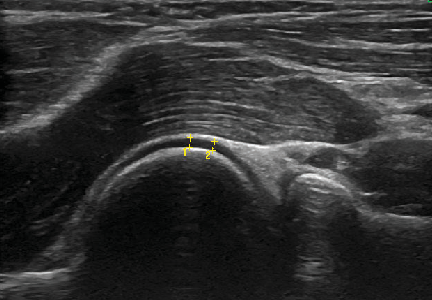


2 L 0.11 cm

1 L 0.11 cm

Apex: 0.11 cm

0.25 cm distal: 0.11 cm


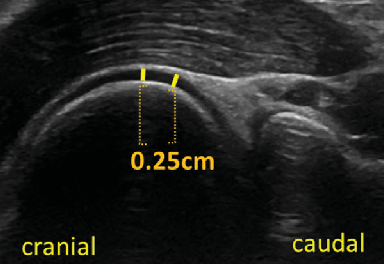
*Example*


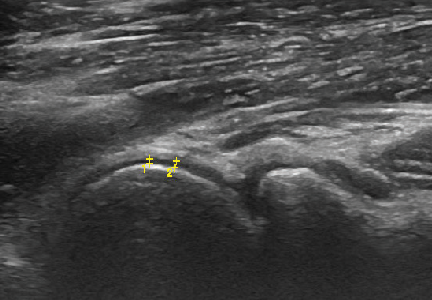


2 L 0.07 cm

1 L 0.05 cm

Apex: 0.05 cm

0.25 cm distal: 0.07 cm


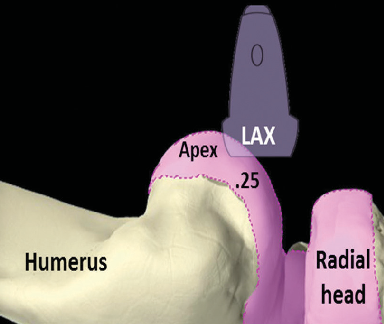

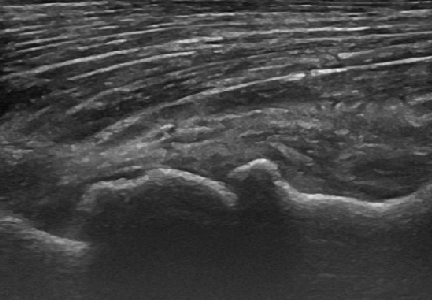
*Example*

Apex: 0.00 cm

0.25 cm distal: 0.00 cm

## Posterior Elbow LAX

Measurement of Content Expansion in the Olecranon Recess and Effusion/Bleed Detection

Patient position: Supine, upper extremity in internal rotation, 90° elbow flexion, and palm resting on abdomen; bolster underneath the elbow will provide support

Axis: LAX

Probe position: Posterior elbow superior to the olecranon

Bony landmarks: Humerus (proximal) and olecranon (distal)

Muscles: Triceps tendon (most superior/attaches to olecranon) and medial triceps belly (inferior to triceps tendon)

Blood vessels: None

Nerves: None

Measurement: Encircle the content of the olecranon fossa, yielding a two-dimensional area.


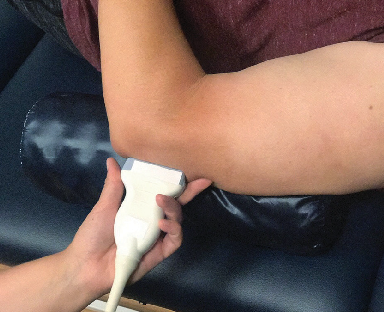
*Example*


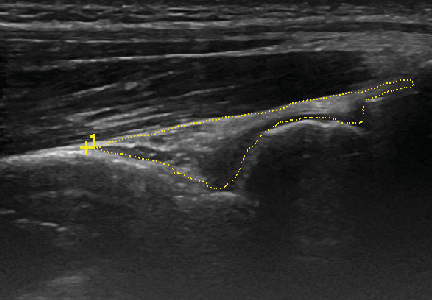


C 8.07 cm

1 A 1.04 cm2

Area: 1.04 cm2


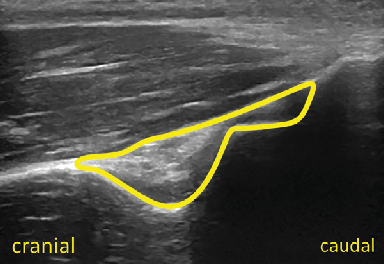
*Example*


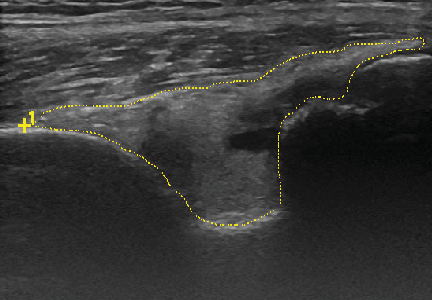


C 11.31 cm

1 A 3.16 cm2

Area: 3.16 cm2


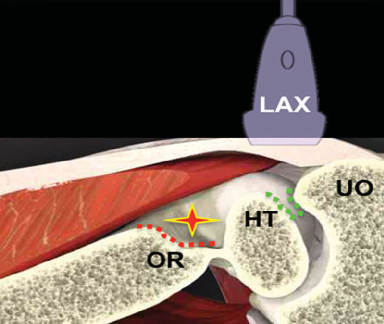
*Example*


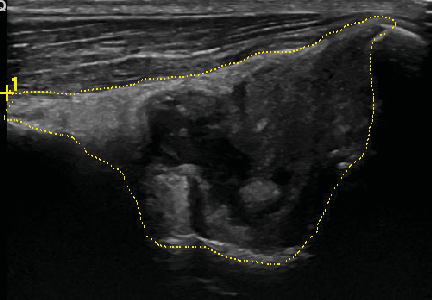


C 12.53 cm

1 A 6.63 cm2

Area: 6.63 cm2


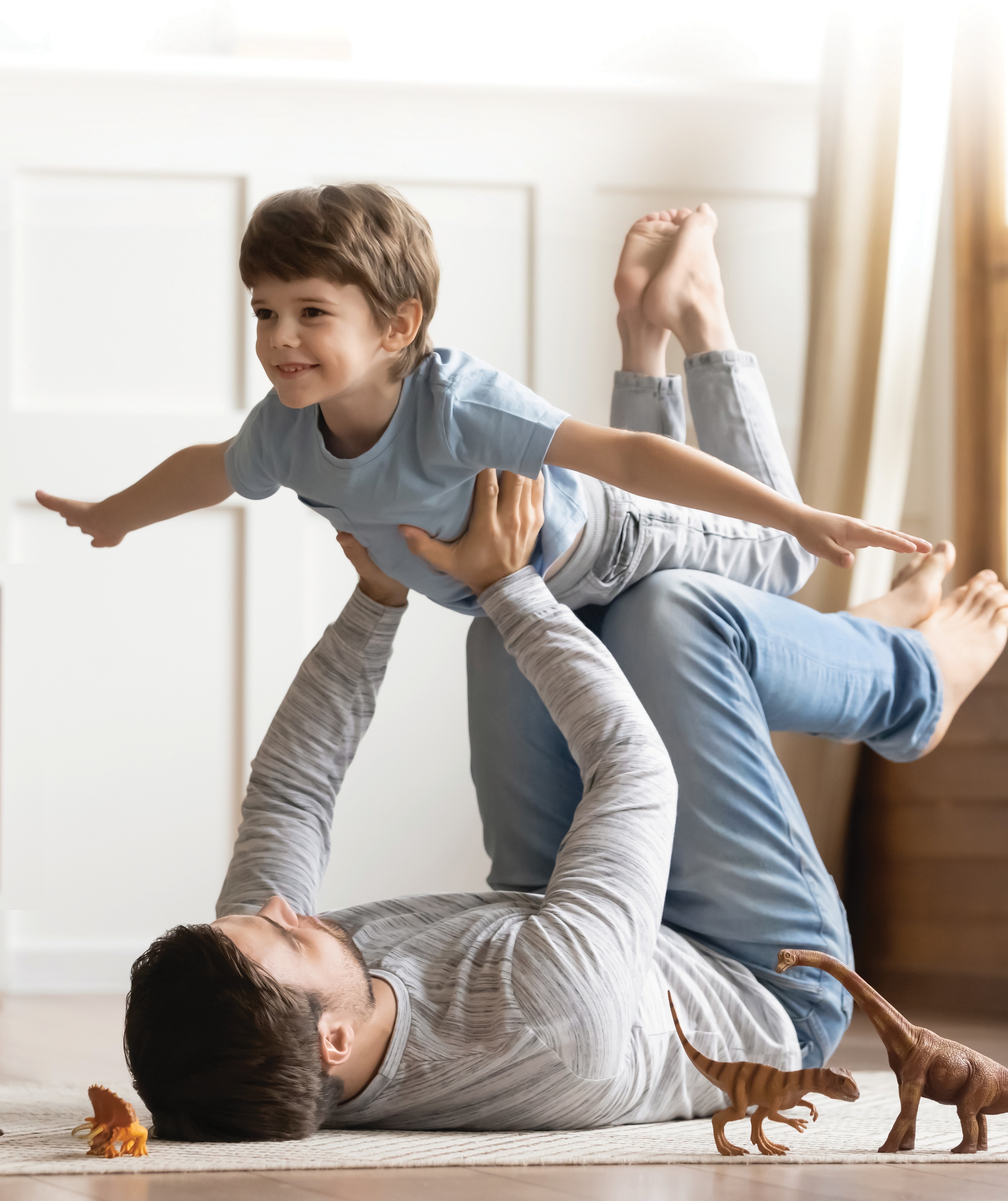


# Knee

10

## Anatomy of the Knee

Joints

- Tibiofemoral: Synovial hinge joint between femur and tibia
- Patellofemoral: Between femur and patella (sesamoid bone)
- Proximal tibiofibular: Arthrodial joint between tibia and fibula

Articulating Surfaces

- Tibiofemoral: Lateral and medial femoral condyle and tibial plateau
- Patellofemoral: Posterior surface of patella and patellar groove of femur
- Proximal tibiofibular: Head of the fibula and lateral tibia

Ligaments

- Extra-capsular: Lateral collateral ligament (LCL), medial collateral ligament (MCL), patella ligament, oblique popliteal ligament, arcuate popliteal ligament
- Intra-capsular: Anterior cruciate ligament (ACL), posterior cruciate ligament (PCL)

Support Structures

- Medial/lateral meniscus

## J.A.D.E. Views

Joint Motion

- Flexion and extension
- Medial/lateral rotation

Associated Muscles

- Flexion: Semimembranosus, semitendinosus, biceps femoris, gastrocnemius, sartorius, gracilis, plantaris, popliteus
- Extension: Quadriceps
- Medial rotation: Semitendinosus, semimembranosus, popliteus, gracilis, sartorius
- Lateral rotation: Biceps femoris

Associated Nerves

- Branches of the femoral, tibial, common peroneal, and obturator nerves

Associated Blood Supply

- Anastomosis of branches of popliteal, femoral, lateral circumflex femoral, circumflex fibular, anterior/posterior tibial recurrent arteries

Bursae

- Superior: Suprapatellar
- Inferior: Prepatellar
- Inferior: Infrapatellar (deep and subcutaneous)
- Medial: Pre anserine
- Posterior: Semimembranosus

Six views are provided for optimal assessment of soft tissue proliferation and inflammation, effusion detection, and osteochondral evaluation. Osteochondral views encompass cartilage health and bony surfaces as well as the osteochondral interface. These standardized transducer position have been divided into recommended and additional views. All obligatory views allow the collection of quantifiable measurements. In daily practice and depending on individual knee pathology the provider may select the most purposeful views to complete a POC exam and to answer a specific question.

| BLEED DETECTION | |  |
| --- | --- | --- |
| Suprapatellar recess | Medial recess Lateral recess | Sunrise |
|  | OBLIGATORY | |

OPTIONAL

Infrapatellar recess Medial meniscus/MCL

## Suprapatellar Recess

Assessment of Content of Suprapatellar Bursa, Surrounding Structures and Effusion/Bleed Detection Patient position: Supine, knee at 30° flexion

Axis: LAX

Probe position: Proximal to the patella and in midline of the knee joint

Bony landmarks: Femur (proximal) and patella (distal)

Muscles: Quadriceps tendon (superior)

Blood Vessels: None

Nerves: None

Other: Suprapatellar bursa and fat pad between femur and quadriceps


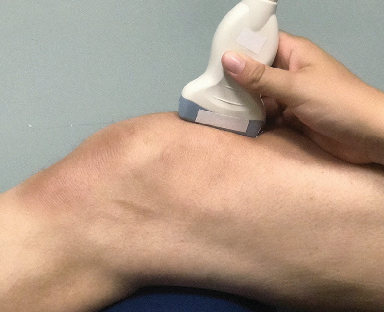
*Example*


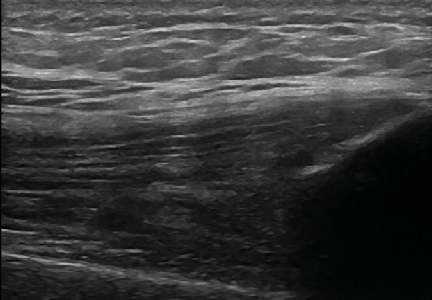

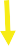


**Suprapatellar bursa**

Normal suprapatellar bursa


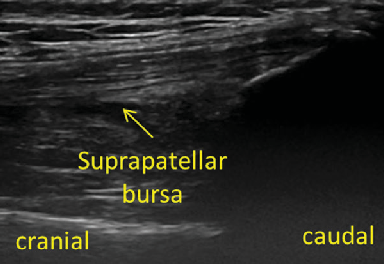
*Example* Suprapatellar bursa with protruding soft


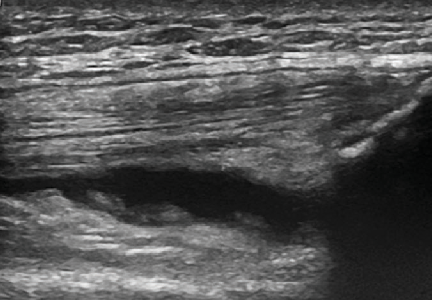

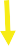


**Suprapatellar bursa**

tissue and compressible anechoic simple effusion


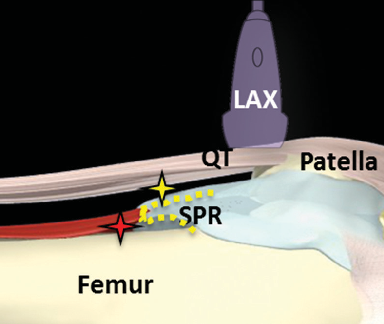
*Example*


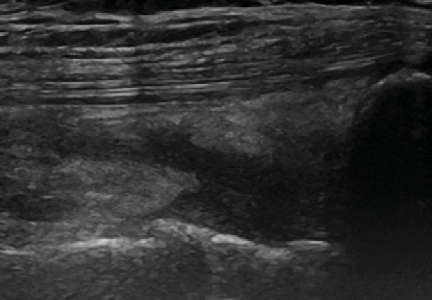

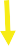


**Suprapatellar bursa**

Suprapatellar bursa with

noncompressible mixed

echogenicity indicative of

soft tissue expansion

## Medial Recess

Measurement of Soft Tissue Expansion and Effusion/Bleed Detection Patient position: Supine, knee at 30° flexion

Axis: SAX

Probe position: Medial mid patella

Bony landmarks: Patella (medial) and femur (lateral)

Muscles: None Blood vessels: None Nerves: None

Other: Retinaculum (medial to patella)

Measurement: Assess soft tissue expansion by measuring the height of the recess at the origin of the femoral cortex and at 0.5 cm and 1.0 cm from the origin of the femoral cortex. Assure that measurement is perpendicular to the bony margin.


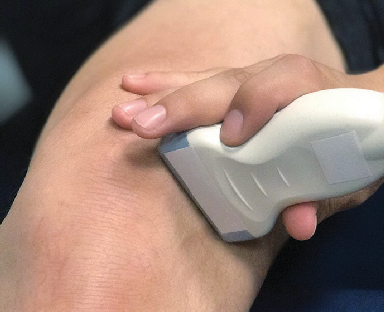
*Example*


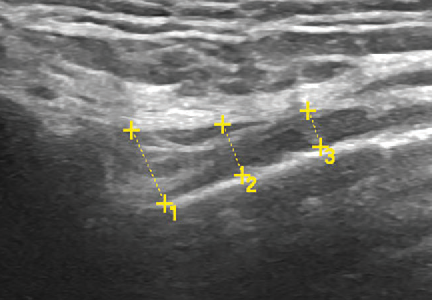


3 L 0.24 cm

2 L 0.33 cm

1 L 0.49 cm

Femoral cortex: 0.49 cm

0.5 cm: 0.33 cm 1 cm: 0.24 cm


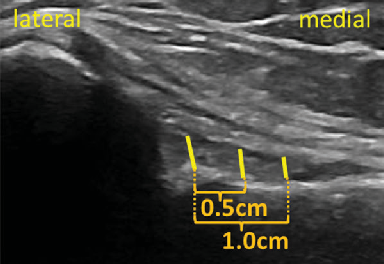
*Example*


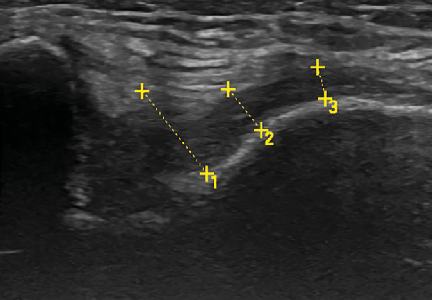


3 L 0.23 cm

2 L 0.37 cm

1 L 0.74 cm

Femoral cortex: 0.74 cm

0.5 cm: 0.37 cm 1 cm: 0.23 cm


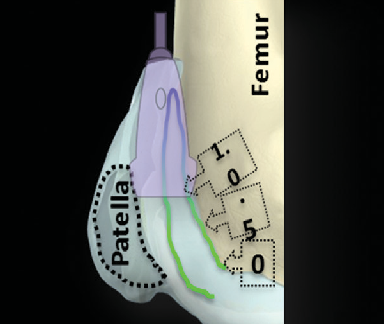
*Example*


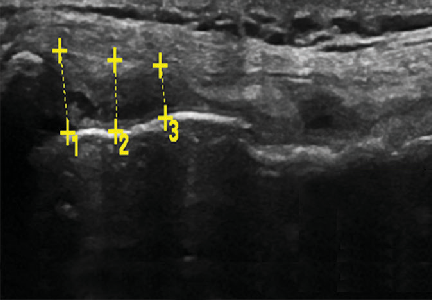


3 L 0.36 cm

2 L 0.48 cm

1 L 0.55 cm

Femoral cortex: 0.55 cm

0.5 cm: 0.48 cm 1 cm: 0.36 cm

## Lateral Recess

Measurement of Soft Tissue Expansion and Effusion/Bleed Detection Patient position: Supine, knee at 30° flexion

Axis: SAX

Probe position: Lateral mid patella

Bony landmarks: Patella (medial) and femur (lateral)

Muscles: None Blood Vessels: None Nerves: None

Other: Retinaculum (lateral to patella)

Measurement: Assess soft tissue expansion by measuring the height of the recess at the origin of the femoral cortex and at 0.5 cm and 1.0 cm from the origin of the femoral cortex. Assure that measurement is perpendicular to the bony margin.


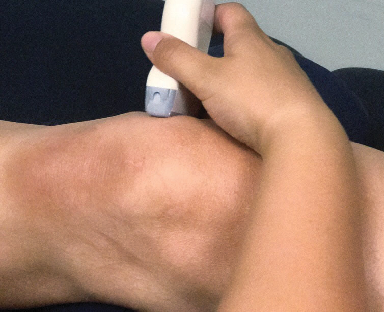
*Example*


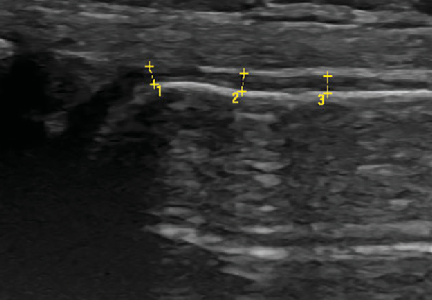


3 L 0.10 cm

2 L 0.11 cm

1 L 0.11 cm

Femoral cortex: 0.11 cm

0.5 cm: 0.11 cm

1.0 cm: 0.10 cm


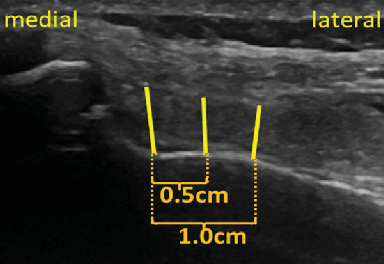
*Example*


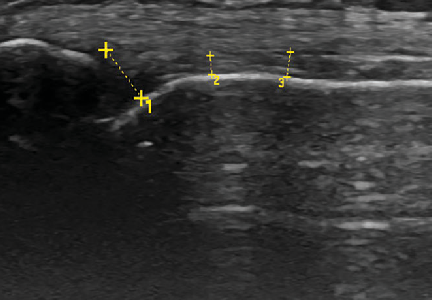


3 L 0.17 cm

2 L 0.13 cm

1 L 0.41 cm

Femoral cortex: 0.41 cm

0.5 cm: 0.13 cm

1.0 cm: 0.17 cm


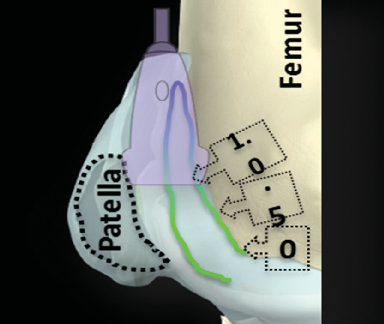
*Example*


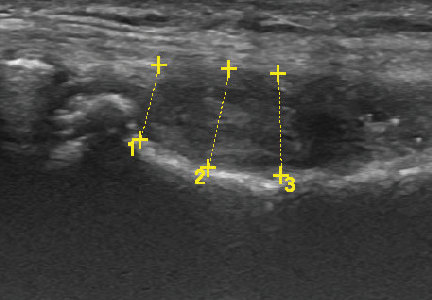


3 L 0.71 cm

2 L 0.71 cm

1 L 0.54 cm

Femoral cortex: 0.54 cm

0.5 cm: 0.71 cm

1.0 cm: 0.71 cm

## Sunrise

Measurement of Osteochondral Interface

Patient position: Supine, knee at 90° flexion, foot planted

Axis: SAX

Probe position: Proximal to patella

Bony landmarks: Femoral trochlea

Muscles: Quadriceps tendon (superior) and vastus medialis (medial)

Blood vessels: None

Nerves: None

Measurement (osteochondral alteration): Individually measure any variation from hyperechoic, smooth, intact bony interface. Then add the values together for the total length of alterations.


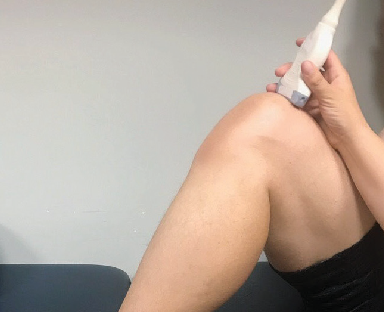
*Example*


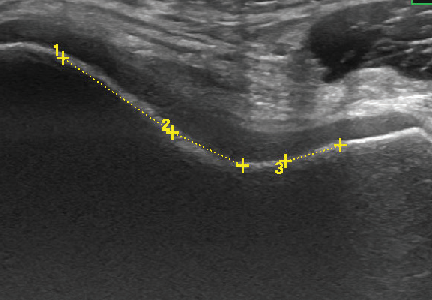


3 L 0.53 cm

2 L 0.73 cm

1 L 1.23 cm

Total length: 2.49 cm


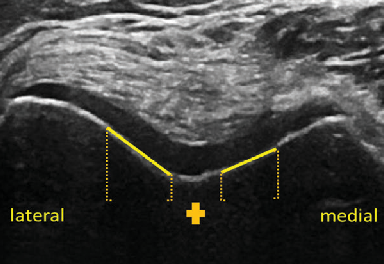
*Example*


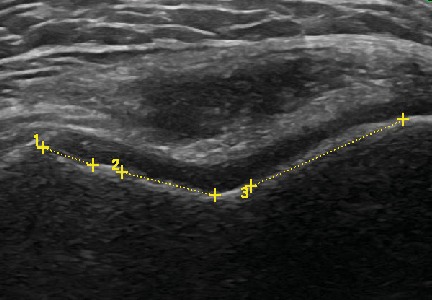


3 L 1.61 cm

2 L 0.93 cm

1 L 0.52 cm

Total length: 3.06 cm


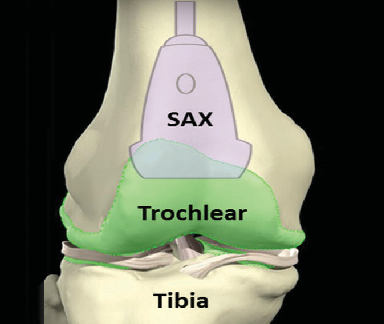
*Example*


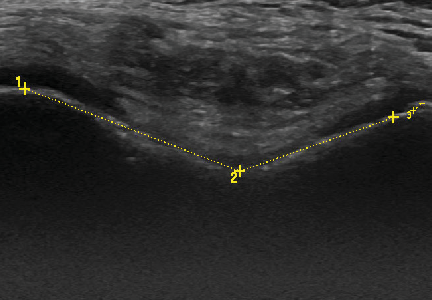


3 L 1.10 cm

2 L 1.47 cm

1 L 2.08 cm

Total length: 3.65 cm

## Sunrise

Measurements of Cartilage Thickness

Patient position: Supine, knee at 90° flexion, foot planted

Axis: SAX

Probe position: Proximal to patella

Bony landmarks: Femoral trochlea

Muscles: Quadriceps tendon (superior) and vastus medialis (medial)

Blood vessels: None

Nerves: None

Measurement (cartilage thickness): Assess cartilage thickness at the lowest point of the trochlea and

0.5 cm medially and laterally. Assure that measurement is perpendicular to the bony margin.


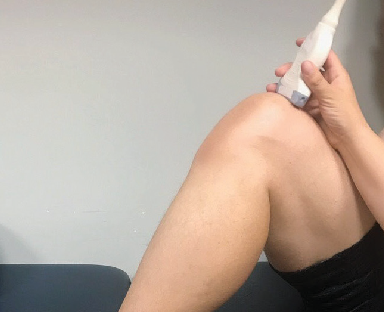
*Example*


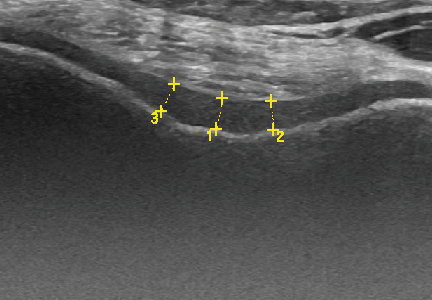


3 L 0.26 cm

2 L 0.25 cm

1 L 0.27 cm

Trochlea: 0.27 cm

0.5 cm medial: 0.25 cm

0.5 cm lateral: 0.26 cm


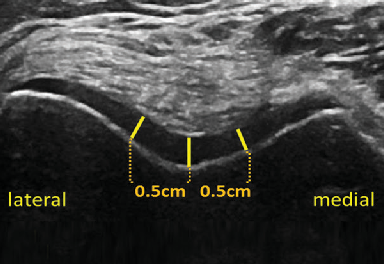
*Example*


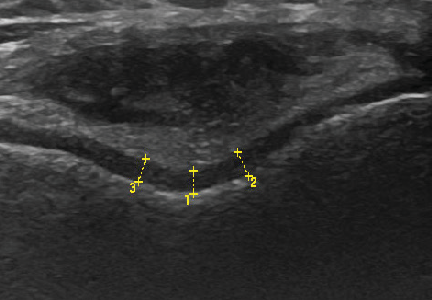


3 L 0.21 cm

2 L 0.23 cm

1 L 0.20 cm

Trochlea: 0.20 cm

0.5 cm medial: 0.23 cm

0.5 cm lateral: 0.21 cm


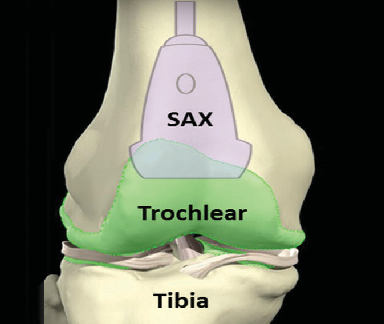
*Example*


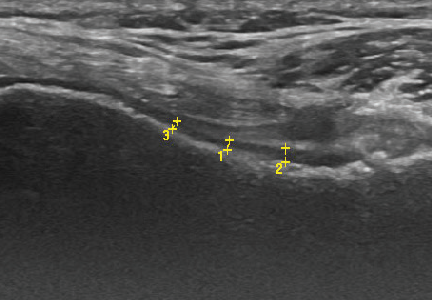


3 L 0.08 cm

2 L 0.12 cm

1 L 0.08 cm

Trochlea: 0.08 cm

0.5 cm medial: 0.12 cm

0.5 cm lateral: 0.08 cm

## Infrapatellar Recess

Assessment of the Hoffa Fat Pad and Surrounding Structures Patient position: Supine, knee at 30° flexion

Axis: LAX

Probe position: Distal to patella

Bony landmarks: Patella (proximal) and tibia (distal) Muscles: Patellar tendon (central between patella and tibia) Blood Vessels: None

Nerves: None

Other: Hoffa fat pad inferior to patellar tendon


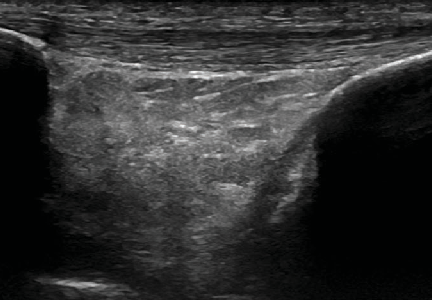

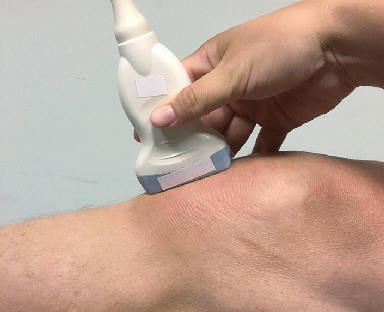
*Example*

Normal patella ligament and Hoffa fat pad


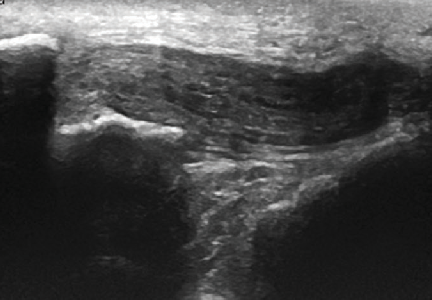

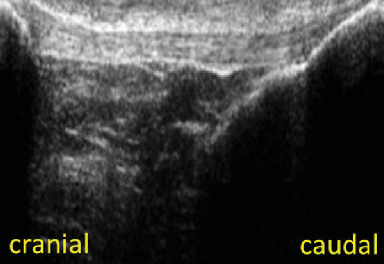
*Example*

Abnormal patella ligament and Hoffa fat pad


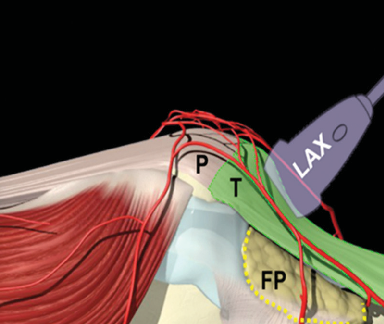

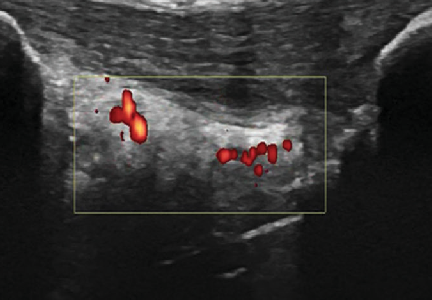
*Example*

Abnormal patella ligament and Hoffa fat pad with PD signal

## Medial Meniscus and MCL

Assessment of Continuity of Medial Meniscus and MCL

Patient position: Supine, knee at 30° flexion, external rotation of the hip

Axis: LAX

Probe position: Medial joint space

Bony landmarks: Femur (proximal) and tibia (distal)

Muscles: None Blood Vessels: None Nerves: None

Other: Medial meniscus (between femur and tibia) and MCL (superior to meniscus)


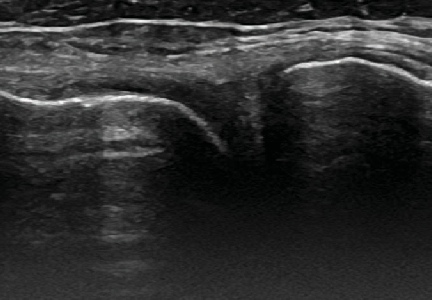

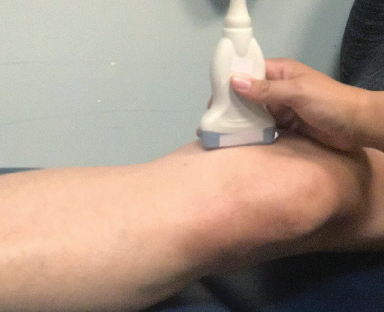
*Example*

Mild changes in echogenicity of meniscus


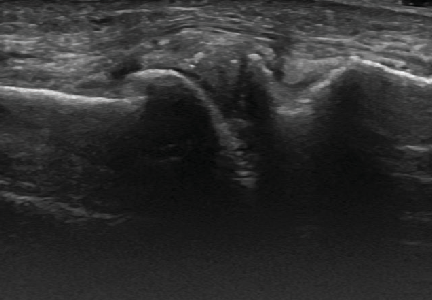

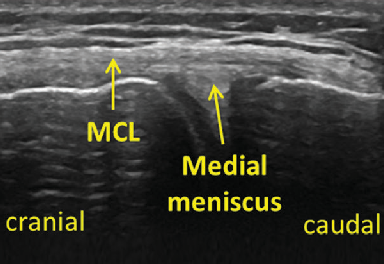
*Example*

Abnormal meniscus echogenicity with changes of regularity in meniscus, bone and MCL


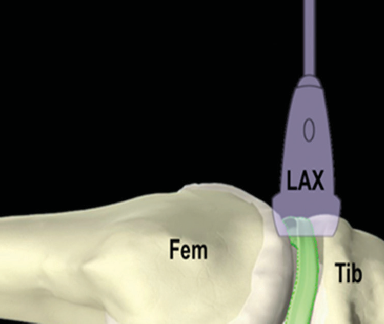

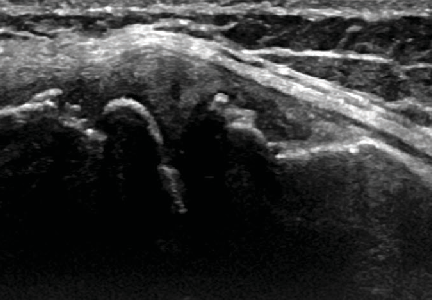
*Example*

Significant irregularities of meniscus, MCL and bone


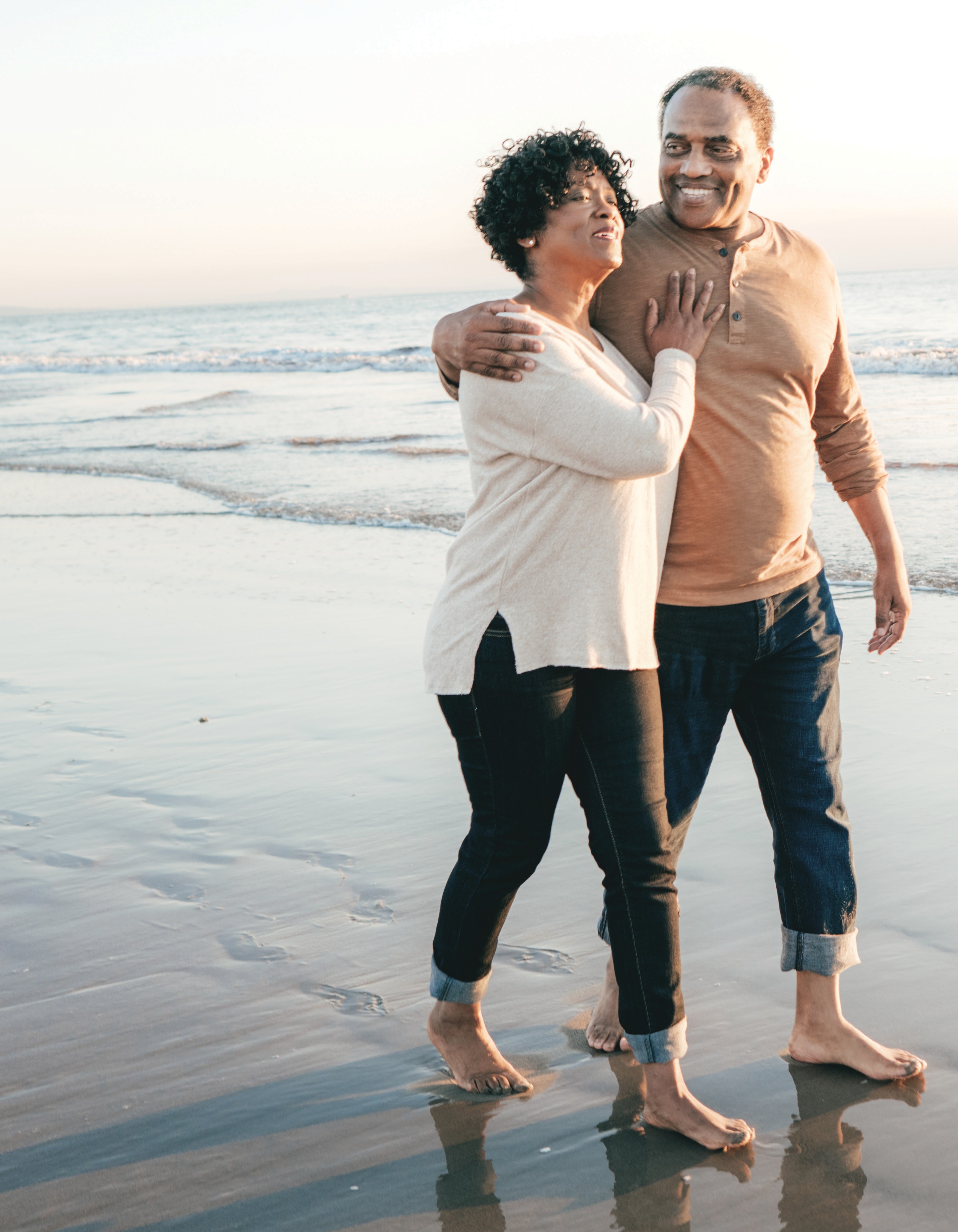


# Ankle

Ultrasonography and Hemophilia | J.A.D.E. Protocol | UC San Diego Health 19

## Anatomy of the Ankle

Joints

- Tibiotalar/talocrual: Hinge type joint between tibia, fibular and talus
- Talocalcaneal/subtalar: Condyloid joint between calcaneus and talus
- Distal tibiofibular: Syndesmosis between distal tibia and fibular

Articulating Surfaces

- Tibiotalar/talocrual: Mortise between fibula and tibia and trochlea of talus
- Subtalar: Three facets of talus and superior surface of calcaneus
- Distal tibifibular: Inferior end of tibia and fibula

Pertinent Ligaments

- Lateral complex: Anterior talofibular ligament (ATFL), calcaneofibular ligament (CFL), and posterior talofibular ligament (PTFL)
- Medial complex/deltoid ligament: Posterior tibiotalar ligament (PTTL), tibiocalcaneal ligament, tibionavicular ligament, anterior tibiotalar ligament
- Anterior: Anterior inferior tibiofibular ligament (AITF)
- Posterior: Posterior inferior tibiofibular ligament (PITF)

Joint Motion

- Tibiotalar: Dorsiflexion and plantarflexion
- Subtalar: Inversion and eversion
- Distal Tibiofibuar: No voluntary motion

Associated Muscles

- Dorsiflexion: Tibialis anterior
- Plantarflexion: Gastrocnemius, soleus, plantaris, tibialis posterior, peroneus longus and brevis
- Inversion: Tibialis posterior
- Eversion: Peroneus longus and brevis

Associated Nerves

- Tibial, sural, saphenous, and deep fibular nerves

Associated Blood Supply

- Branches of the anterior tibial (dorsalis pedis), posterior tibial and fibular arteries

## J.A.D.E. Views

Four views are provided for optimal assessment of soft tissue proliferation and inflammation, effusion detection, and osteochondral evaluation. Additionally, the ankle includes an assessment of the integrity of the Achilles tendon. It is important to note that the ankle has an extra-synovial, but intra-capsular fat pad yielding a combined interface for measurement according to the J.A.D.E. protocol. The four standardized transducer positions are divided into two obligatory and two optional views. However, the provider

may find it useful to select the most purposeful transducer positions for POC exams depending on the underlying ankle pathology.

| BLEED DETECTION | |  |
| --- | --- | --- |
|  | Anterior ankle SAX Anterior ankle LAX |  |
|  | OBLIGATORY | |

OPTIONAL

Achilles Subtalar


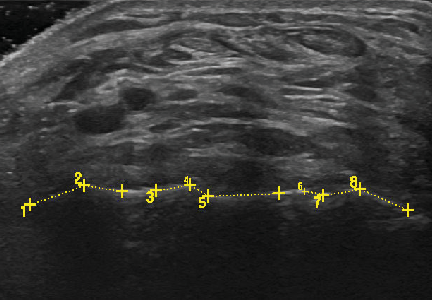


## Anterior Ankle SAX

Measurement of Osteochondral Interface Irregularities and Effusion/Bleed Detection Patient position: Supine, foot rests on bolster

Axis: SAX

Probe position: Across anterior tibiotalar joint

Bony landmarks: Talus

Muscles: Superior to tibia, extensor digitorum longus tendon, extensor hallucis tendon, tibialis anterior tendon

Blood vessels: Dorsalis pedis artery and vein

Nerves: Deep peroneal nerve

Measurement (osteochondral alteration): Individually measure any variation from hyperechoic, smooth, intact bony interface. Then add the values together for the total length of alterations.


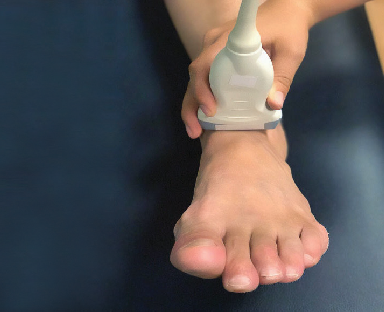
*Example*


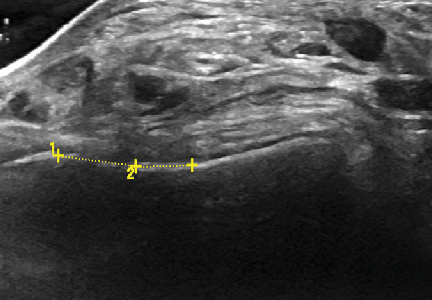


2 L 0.51 cm

1 L 0.71 cm

Total length: 1.22 cm

*Example*

Total length: 1.85 cm

| 1 L 0.49 cm |
| --- |
| 2 L 0.44 cm |
| 3 L 0.13 cm |
| 4 L 0.79 cm |

*Example*

| 1 L 0.57 cm |
| --- |
| 2 L 0.38 cm |
| 3 L 0.34 cm |
| 4 L 0.21 cm |
| 5 L 0.71 cm |
| 6 L 0.19 cm |
| 7 L 0.37 cm |
| 8 L 0.52 cm |

Total length: 3.63 cm

## Anterior Ankle SAX

Measurement of Capsular Interface Thickness and Effusion/Bleed Detection Patient position: Supine, foot rests on bolster

Axis: SAX

Probe position: Across anterior tibiotalar joint

Bony landmarks: Talus

Muscles: Superior to tibia, extensor digitorum longus tendon, extensor hallucis tendon, tibialis anterior tendon

Blood vessels: Dorsalis pedis artery and vein

Nerves: Deep peroneal nerve

Measurement (capsular interface): Measure the thickness of the capsular interface from the beginning of the synovial membrane to the height of the dorsalis pedis artery muscle at the lowest point of the trochlea and 0.5 cm to the medial and lateral side. Assure that measurement is perpendicular to the bony margin.

*Example*

3 L 0.51 cm

2 L 0.57 cm

1 L 0.55 cm

Trochlea: 0.55 cm

0.5 cm medial: 0.57 cm

0.5 cm lateral: 0.51 cm

*Example*

3 L 0.67 cm

2 L 0.65 cm

1 L 0.67 cm

Trochlea: 0.67 cm

0.5 cm medial: 0.65 cm

0.5 cm lateral: 0.67 cm

*Example*

3 L 0.94 cm

2 L 0.99 cm

1 L 1.05 cm

Trochlea: 1.05 cm

0.5 cm medial: 0.99 cm

0.5 cm lateral: 0.94 cm

## Anterior Ankle LAX

Measurement of Cartilage Thickness and Effusion/Bleed Detection Patient position: Supine, foot rests on bolster

Axis: LAX

Probe position: At the midline of the anterior talocrural joint

Bony landmarks: Tibia (proximal), talar dome (center), and talar head (distal)

Muscles: Extensor digitorum longus tendon (superficial) Blood vessels: Dorsalis pedis artery (deep to tendon) Nerves: None

Other: Fat pad anterior to talocrural joint space

Measurement: Assess cartilage thickness perpendicularly to the bony margin at the apex and

0.25 cm proximally.

*Example*

2 L 0.08 cm

1 L 0.08 cm

Apex: 0.08 cm

0.25 cm proximal:

0.08 cm

*Example*

2 L 0.05 cm

1 L 0.04 cm

Apex: 0.04cm

0.25 cm proximal:

0.05 cm

*Example*

Apex: 0.00cm

0.25 cm proximal:

0.00 cm

## Achilles Tendon

Measurement of Achilles Tendon Thickness Patient position: Prone, foot resting on a bolster

Axis: LAX

Probe position: Distal Achilles tendon

Bony landmarks: Tibia (proximal) and calcaneus (distal)

Muscles: Achilles tendon (superior), tibialis posterior tendon (superior to tibia), and flexor digitorum tendon (superior to tibialis posterior)

Blood vessels: None

Nerves: None

Other: Kager’s fat pad (deep to Achilles tendon), retro-calcaneal bursa (between Achilles tendon and calcaneus)

Measurement (capsular interface): Assess tendon thickness by measuring perpendicularly at 2 cm proximally from the end of the calcaneus.

*Example*

1 L 0.47 cm

2 cm from calcaneus:

0.47 cm

*Example*

1 L 0.58 cm

2 cm from calcaneus:

0.58 cm

*Example*

1 L 0.68 cm

2 cm from calcaneus:

0.68 cm

## Subtalar Joint

Assessment of Subtalar Joint Abnormalities

Patient position: Supine, foot rests on bolster, external rotation of the hip

Axis: LAX

Probe position: Connecting medial malleolus with calcaneus

Bony landmarks: Medial malleolus (proximal), sustentaculum tali (center), calcaneus (distal)

Muscles: None Blood vessels: None Nerves: None

*Example*

**Subtalar joint space**

Mild irregularities

*Example*

**Subtalar joint space**

Irregular bony margin

*Example*

**Subtalar joint space**

Irregular bony margin and soft tissue expansion

# About the Authors

## Annette von Drygalski, MD, PharmD, RMSK

Annette von Drygalski is a Professor of Clinical Medicine in the Division of Hematology/Oncology, and Director of the Hemophilia and Thrombosis Treatment Center, Department of Medicine, at the University of California, San Diego.

Dr. von Drygalski earned her pharmaceutical and medical degrees from the Ludwig Maximilians University, Munich, Germany. She completed residency in Internal Medicine at the Medical College of Wisconsin, Milwaukee, and fellowship in

Hematology and Oncology at the University of California San Diego and The Scripps Research Institute.

Dr. von Drygalski is dedicated to the care of patients with hemophilia and other bleeding disorders addressing the evolving needs of aging patients with hemophilia, such as new therapies, cardiovascular disease and hypertension as well as the progression of hemophilic joint disease. In this role, Dr. von Drygalski investigates novel molecules in clinical trials, and elucidates effects of new treatments on the pathobiology of joint health, both at the bench and in translational studies. Dr. von Drygalski has pioneered musculoskeletal ultrasound for rapid joint bleed detection and joint evaluation in hemophilia. She is certified in musculoskeletal ultrasound by the American Registry for Diagnostic Medical Sonography, and has developed the Joint Activity and Damage Exam (J.A.D.E.), a validated ultrasound protocol to quantify synovial and osteochondral changes as well as inflammation in the hemophilic joint. She is also the Director of the CME-accredited course “Musculoskeletal Ultrasound in Hemophilia” at the University of California San Diego.

More recently Dr. von Drygalski developed a focus in international outreach to afford training for hematologists from developing countries and establish care for patients with hemophilia and bleeding disorders in Mozambique, as well as address mortality from post-partum bleeding.

## Randy Moore, DC, RDMS, RMSK

Dr. Moore is a highly regarded and sought after pioneer, teacher and author in the quickly emerging field of musculoskeletal sonography. His straightforward, easily understood teaching methods have been deemed essential to shortening the learning curve for medical practitioners, medical sonographers, physician assistants, nurse practitioners, physical therapists and allied health professionals as they develop expertise in MSK sonography.

Worldwide, more and more healthcare professionals are tapping the well of diagnostic sonography — it is quickly becoming the standard of care for visualizing musculoskeletal anatomy.

Randy E. Moore is a Doctor of Chiropractic and a Registered Diagnostic Medical Sonographer. Since 1994, his work has focused entirely on using diagnostic ultrasound to evaluate the musculoskeletal system. His expertise is called upon by ultrasound manufacturers (GE, Sonosite, Ultrasonix, Esaote, Terason, Phillips) and teaching institutions (National Institute of Health, Beth Israel Hospital NY, Walter Reed, University of Dayton, University of Kentucky, Temple, and Loma Linda) throughout the United States.

## Lena Volland, PT, DPT, OCS

Lena Volland is the Director of Education at the National Hemophilia Foundation (NHF). Born and raised in Germany, she received her initial education in Europe and further enhanced her knowledge with degrees in kinesiology and physical therapy in the United States. Prior to joining NHF, she was a full-time faculty member at the University of St. Augustine in their Doctor Physical Therapy program, where she taught imaging and orthopedic treatment courses. Preceding this role, she worked as physical therapist in clinical care and research at the UCSD Hemophilia and

Thrombosis Treatment Center. She has been substantially involved in the validation process of the Joint Activity and Damage Exam (J.A.D.E.) ultrasound protocol and has been a faculty member of the CME accredited course “Musculoskeletal Ultrasound in Hemophilia” at the University of California San Diego. She is currently pursuing a PhD in Translational Health Sciences at George Washington University with a focus on chronic pain management in people with bleeding disorders.

## Eric Chang, MD

Eric Chang, MD, is a staff physician at the VA San Diego Healthcare System and professor of radiology at the University of California San Diego Medical Center. He is also the director of the San Diego VA/Veterans Medical Research Foundation Microimaging Core which provides preclinical imaging services to the regional research community, including histology, electron microscopy, and in vivo optical,

ultrasound (US), micro-computed tomography and magnetic resonance (MR) imaging.

Dr. Chang completed his undergraduate and medical studies at Cornell University (1998-2001) and New York University School of Medicine (2001-2005), respectively. He completed his residency and musculoskeletal radiology fellowship at UC San Diego (2006-2011), where he was also chief resident. Dr. Chang was bestowed the Presidential Early Career Awards for Scientists and Engineers (PECASE) award in 2017, which is the highest honor given by the United States government to science and engineering professionals in the early stages of their independent research careers.

Dr. Chang’s research lab investigates imaging technologies, including those to assess the microstructure, composition, and chemical environment of neuromusculoskeletal tissues, focusing on quantitative MR and US imaging. He has authored over 200 peer-reviewed publications and is an active member of numerous societies, including the American College of Radiology where he serves as the specialty chair on the Appropriateness Criteria Musculoskeletal Imaging section and International Prophylaxis Study Group where he serves as a member of the Imaging Working Group. Dr. Chang is the current Musculoskeletal Section editor for the *American Journal of Roentgenology*, which is recognized as among the specialty's leading

peer-reviewed journals.

## Peter Aguero, PT, DPT, RMSK

Peter Aguero is the specialized Doctor of Physical Therapy of the Hemophilia and Thrombosis Treatment Center at the University of California, San Diego. In addition, he is a senior physical therapist for the Department of Rehabilitation at the Jacobs Medical Center and Thornton Hospital in La Jolla, California. He earned his Doctor of Physical Therapy at the University of St. Augustine for Health Sciences in San Marcos, California.

Dr. Aguero is committed to improving the overall health and wellness of patients with hemophilia and the treatment of associated impairments such as joint arthropathy, muscle bleeds, heterotopic ossifications, chronic pain and consequential gait/movement deviations. He was involved with researching the validation of the Musculoskeletal Ultrasound Joint Activity and Damage Exam (J.A.D.E.) protocol, the development of wellness/exercise programs for this patient population and researching the feasibility of patient self- imaging with ultrasound via teleguidance. In addition, he is the co-director for the CME-accredited course “Musculoskeletal Ultrasound Training for Hemophilia and Other Arthritic Conditions” and has earned his Registered in Musculoskeletal sonography certification.

*Special thanks to the team and patients of the UC San Diego Health Hemophilia and Thrombosis Treatment Center for their continuous support and contributions.*

# References

Bakeer N, Dover S, Babyn P, Feldman BM, von Drygalski A, Doria AS, Ignas DM, Abad A, Bailey C, Beggs I, Chang EY, Dunn A, Funk S, Gibikote S, Goddard N, Hilliard P, Keshava SN, Kruse-Jarres R, Li Y, Lobet S, Manco-Johnson M, Martinoli C, O'Donnell JS, Papakonstantinou O, Pergantou H, Poonnoose P, Querol F, Srivastava A, Steiner B, Strike K, Timmer M, Tyrrell PN, Vidarsson L,

Blanchette VS. Musculoskeletal ultrasound in hemophilia: Results and recommendations from a global survey and consensus meeting. Res Pract Thromb Haemost. 2021 Jul 10;5(5):e12531. doi: 10.1002/rth2.12531. PMID: 34268464; PMCID: PMC8271584.

Mesleh Shayeb A, Barnes RFW, Hanacek C, Aguero P, Steiner B, Bailey C, Quon D, Kruse-Jarres R, von Drygalski A. Quantitative measurements of haemophilic joint tissues by point-of-care musculoskeletal ultrasound: Associations with clinical and functional joint outcome parameters. Haemophilia. 2021 Sep;27(5):866-875. doi: 10.1111/hae.14368. Epub 2021 Jun 25. PMID: 34171150.

Ignas DM, Doria AS, von Drygalski A, Blanchette VS, Chang EY, Dover S, Fischer K, Gibikote S, Keshava SN, Querol F, Abad A, Babyn

P. Use of ultrasound for assessment of musculoskeletal disease in persons with haemophilia: Results of an International Prophylaxis Study Group global survey. Haemophilia. 2020 Jul;26(4):685-693. doi: 10.1111/hae.14006. Epub 2020 May 22. PMID: 32441402.

von Drygalski A, Moore RE, Nguyen S, Barnes RFW, Volland LM, Hughes TH, Du J, Chang EY. Advanced Hemophilic Arthropathy: Sensitivity of Soft Tissue Discrimination With Musculoskeletal Ultrasound. J Ultrasound Med. 2018 Aug;37(8):1945-1956. doi: 10.1002/jum.14541. Epub 2018 Jan 24. PMID: 29363781; PMCID: PMC6057843.

Nguyen S, Lu X, Ma Y, Du J, Chang EY, von Drygalski A. Musculoskeletal ultrasound for intra-articular bleed detection: a highly sensitive imaging modality compared with conventional magnetic resonance imaging. J Thromb Haemost. 2018 Mar;16(3):490-499. doi: 10.1111/jth.13930. Epub 2018 Jan 19. PMID: 29274196; PMCID: PMC5826858.

Volland LM, Zhou JY, Barnes RFW, Kruse-Jarres R, Steiner B, Quon DV, Bailey C, Hughes TH, Moore RE, Chang EY, von Drygalski A. Development and Reliability of the Joint Tissue Activity and Damage Examination for Quantitation of Structural Abnormalities by Musculoskeletal Ultrasound in Hemophilic Joints. J Ultrasound Med. 2019 Jun;38(6):1569-1581. doi: 10.1002/jum.14846. Epub 2018 Oct 29. PMID: 30371941.

Moeller I, Janta I, Backhaus M, Ohrndorf S, Bong DA, Martinoli C et al. The 2017 EULAR standardized procedures for ultrasound imaging in rheumatology. *Ann Rheum Dis.* Advanced online publication. Doi:10.1136/annrheumdis-2017-211585.

Kidder W, Chang EY, Moran CM, von Drygalski A. Persistent vascular remodeling and leakiness are important components of the pathobiology of re-bleeding in hemophilic joints: Two informative cases. *Mircocirculation* 2016;23(5):373-8.

Kidder W, Chang EY, Moran C, Rose S, and von Drygalski A. Propagation of Hemophilic Arthropathy - the Role of Abnormal Angiogenesis and Vascular Remodeling for Recurrent Joint Bleeding. *Microcirculation*. 2016 Jul;23(5):373-8. doi: 10.1111/micc.12273

Bhat V, Olmer M, Joshi S, Durden DL, Cramer TJ, Barnes RF, Ball ST, Hughes TH, Silva M, Luck JV, Moore RE, Mosnier LO, von Drygalski A. Vascular remodeling underlies rebleeding in hemophilic arthropathy. *Am J Hematol* 2015;90(11):1027-35.

Kidder W, Nguyen S, Larios J, Bergstrom J, Ceponis A, von Drygalski A. Point-of-care musculoskeletal ultrasound is critical for the diagnosis of hemarthroses, inflammation and soft tissue abnormalities in adult patients with painful haemophilic arthropathy. Haemophilia. 2015 Jul;21(4):530-7. doi: 10.1111/hae.12637. Epub 2015 Jan 27. PMID: 25623830.

Ceponis A, Wong-Sefidan I, Glass CS, von Drygalski A. Rapid musculoskeletal ultrasound for painful episodes in adult haemophilia patients. Haemophilia. 2013 Sep;19(5):790-8. doi: 10.1111/hae.12175. Epub 2013 May 15. PMID: 23672827.

Martinoli C, Della Casa Alberighi O, Di Minno G, Graziano E, Molinari AC, Pasta G, Russo G, Santagostino E, Tagliaferri A, Morfini M. Development and definition of a simplified scanning procedure and scoring method for Haemophilia Early Arthropathy Detection with Ultrasound (HEAD-US). *Thromb Haemost* 2013; 109 (6): 1170–9.

Querol F and Rodriguez-Merchan EC. The role of ultrasonography in the diagnosis of the musculskeletal problems of haemophilia.

*Haemophilia* 2012; 18 (3): 215-26.

Ludin B, Manco-Johnson ML, Ignas DM, Moineddin R, Blanchette VS, Dunn AL, Gibikote SV, Keshava SN, Ljung R, Manco-Johnson MJ, Miller SF, Rivard GE, Doria AS. An MRI scale for assessment of haemophilic arthropathy from the International Prophylaxis study group. *Haemophilia* 2012;18(6):962-70.

Backhaus M, Ohrndorf S, Kellner H, Strunk J, Backhaus TM, Hartung W, Sattler H, Albrecht K, Kaufmann J, Becker K, Soerensen H, Meier L, Burmester GR, Schmidt WA. Evaluation of a novel 7-joint ultrasound score in daily rheumatologic practice: A pilot project. *Arthritis Rheum* 2009;61(9):1194-201.

Hillard P, Funk S, Zourikian N, Bergstrom BM, Bradley CS, McLimont M, Manco-Johnson M, Petrini P, van den Berg M, Feldman BM. Hemophilia joint health score reliability study. *Haemophilia* 2006;1(5):518-25.

Wakefield RJ, Balint PV, Szkudlarek M, Filippucci E, Backhaus M, D’Agostino MA, et al. Musculoskeletal ultrasound including definitions for ultrasonographic pathology. *J Rheumatol* 2005; 32(12): 2485-7.

Backhaus M, Burmester GR, Gerber T, Grassi W, Machold KP, Swen WA, Wakefield RJ, Manger B. Guidelines for musculoskeletal ultrasound in rheumatology. *Ann Rheum Dis* 2001; 60(7): 641–9.

Pettersson H, Ahlberg A, Nilsson IM. A radiologic classification of hemophilic arthropathy. *Clin Orthop* 1980;149:153–9.

© 2021 The Regents of the University of California. All rights reserved. Permission to make commercial use of this protocol may be obtained by contacting: Office of Innovation and Commercialization, 9500 Gilman Drive, Mail Code 0910, University of California San Diego, La Jolla, CA 92093-0910 / (858) 534-5815 / [invent@ucsd.edu](mailto:invent@ucsd.edu)

UC San Diego Health – La Jolla

Hemophilia and Thrombosis Treatment Center 9333 Genesee Ave., Suite 310

La Jolla, CA 92121 T: 858-657-6028

F: 858-249-2519

UC San Diego Health – Murrieta

Hemophilia and Thrombosis Treatment Center 41011 California Oaks Road, Suite 104

Murrieta, CA 92562

T: 951-303-0734

F: 951-303-8591

health.ucsd.edu/specialties/blood-disorders/ hemophilia-thrombosis

D6675

JADE ASSESSMENT

Elbow – Anterior Osteochondral Interface: *Osteochondral Line*

Patient name: MR number: Person completing this form: Date:

Joint Space Content

none anechoic hypoechoic mixed signal

Assessment

hypoechoic complex fluid anechoic simple fluid soft tissue proliferation irregular bony margin obliterated joint space osteophytes

other:

Measurement

Length of osteochondral alterations:

Power Doppler (see last page)

1 2 3 4

Compressibility

fully partially non-compressible N/A

Patient position: Supine, palm up with bolster under the hand to limit elbow extension

Axis: SAX

Probe position: In antecubital fossa

Bony landmarks: Ulnar trochlea (medial) and humeral capitulum (lateral)

Muscles: Brachialis (central), brachioradialis (lateral), pronator (medial), biceps brachialis tendon (superficial to brachialis)

Blood vessels: Brachial artery and vein

Nerves: Radial nerve (lateral), median nerve (medial)

Measurement: Individually measure any variation from hyperechoic, smooth, intact bony interface. Then add the values together for the total length of alterations.

Elbow – Anterior Lateral Joint: *Cartilage Thickness*

Patient name: MR number: Person completing this form: Date:

Joint Space Content

none anechoic hypoechoic mixed signal

Assessment

hypoechoic complex fluid anechoic simple fluid soft tissue proliferation

irregular bony margin other:

Measurement

Cartilage thickness apex: 0.25 cm distal to apex:

Power Doppler (see last page)

1 2 3 4

Compressibility

fully partially non-compressible N/A

Patient position: Supine, palm up with bolster under the hand to limit elbow extension

Axis: LAX

Probe position: Lateral aspect of the antecubital fossa over the humeroradial joint space

Bony landmarks: Humeral capitulum (proximal) and radial head (distal)

Muscles: Brachioradialis (superficial to humeroradial joint)

Blood vessels: None

Nerves: None

Measurement: Assess cartilage thickness at the apex and 0.25 cm distally. Assure that measurement is perpendicular to the bony margin.

Elbow – Posterior Joint: *Fat Pad Triceps Tendon*

Patient name: MR number: Person completing this form: Date:

Fat Pad Content

none anechoic hypoechoic mixed signal

Assessment

hypoechoic complex fluid anechoic simple fluid displacement of fat pad tendon abnormality fat pad change of echogenicity

other:

Measurement

Area cm2:

Power Doppler (see last page)

1 2 3 4

Compressibility

fully partially non-compressible N/A

Patient position: Supine, upper extremity in internal rotation, 90° elbow flexion, and palm resting on abdomen; bolster underneath the elbow will provide support

Axis: LAX

Probe position: Posterior elbow superior to the olecranon

Bony landmarks: Humerus (proximal) and olecranon (distal)

Muscles: Triceps tendon (most superior/attaches to olecranon) and medial triceps belly (inferior to triceps tendon)

Blood vessels: None

Nerves: None

Measurement: Encircle the content of the olecranon fossa, yielding a two-dimensional area.

Knee – Suprapatellar Recess: *Suprapatellar Bursa and Structures*

Patient name: MR number: Person completing this form: Date:

Suprapatellar Bursa Content

none anechoic hypoechoic mixed signal

Assessment

hypoechoic complex fluid anechoic simple fluid soft tissue proliferation

tendon abnormality other:

Power Doppler (see last page)

1 2 3 4

Compressibility

fully partially non-compressible N/A

Patient position: Supine, knee at 30° flexion

Axis: LAX

Probe position: Proximal to the patella and in midline of the knee joint

Bony landmarks: Femur (proximal) and patella (distal)

Muscles: Quadriceps tendon (superior)

Blood Vessels: None

Nerves: None

Other: Suprapatellar bursa and fat pad between femur and quadriceps

Knee – Medial Recess: *Soft Tissue Expansion*

Patient name: MR number: Person completing this form: Date:

Medial Recess Content

none anechoic hypoechoic mixed signal

Assessment

hypoechoic complex fluid anechoic simple fluid soft tissue proliferation

irregular bony margin other:

Measurement

Soft tissue measurement during compression: Femoral cortex (start at cortical bone) 0 cm: 0.5 cm: 1.0 cm:

Power Doppler (see last page)

1 2 3 4

Compressibility

fully partially non-compressible N/A

Patient position: Supine, knee at 30° flexion

Axis: SAX

Probe position: Medial mid patella

Bony landmarks: Patella (medial) and femur (lateral)

Muscles: None Blood vessels: None Nerves: None

Other: Retinaculum (medial to patella)

Measurement: Assess soft tissue expansion by measuring the height of the recess at the origin of the femoral cortex and at 0.5 cm and 1.0 cm from the origin of the femoral cortex. Assure that measurement is perpendicular to the bony margin.

Knee – Lateral Recess: *Soft Tissue Expansion*

Patient name: MR number: Person completing this form: Date:

Lateral Recess Content

none anechoic hypoechoic mixed signal

Assessment

hypoechoic complex fluid anechoic simple fluid soft tissue proliferation

irregular bony margin other:

Measurement

Soft tissue measurement during compression: Femoral cortex (start at cortical bone) 0 cm: 0.5 cm: 1.0 cm:

Power Doppler (see last page)

1 2 3 4

Compressibility

fully partially non-compressible N/A

Patient position: Supine, knee at 30° flexion

Axis: SAX

Probe position: Lateral mid patella

Bony landmarks: Patella (medial) and femur (lateral)

Muscles: None Blood Vessels: None Nerves: None

Other: Retinaculum (lateral to patella)

Measurement: Assess soft tissue expansion by measuring the height of the recess at the origin of the femoral cortex and at 0.5 cm and 1.0 cm from the origin of the femoral cortex. Assure that measurement is perpendicular to the bony margin.

Knee – Sunrise: *Osteochondral Line Cartilage Thickness*

Patient name: MR number: Person completing this form: Date:

Assessment

irregular bony margin osteophytes other:

Measurement

Length of osteochondral alterations: Cartilage thickness trochlea 0.5 cm medial: 0.5 cm lateral:

Power Doppler (see last page)

1 2 3 4

Compressibility

fully partially non-compressible N/A

Patient position: Supine, knee at 90° flexion, foot planted

Axis: SAX

Probe position: Proximal to patella

Bony landmarks: Femoral trochlea

Muscles: Quadriceps tendon (superior) and vastus medialis (medial)

Blood vessels: None

Nerves: None

Measurement (osteochondral alteration): Individually measure any variation from hyperechoic, smooth, intact bony interface. Then add the values together for the total length of alterations.

Measurement (cartilage thickness): Assess cartilage thickness at the lowest point of the trochlea and 0.5 cm medially and laterally. Assure that measurement is perpendicular to the bony margin.

Knee – Infrapatellar Recess: *Hoffa Fat Pad*

Patient name: MR number: Person completing this form: Date:

Infrapatellar Fat Pad Content

none anechoic hypoechoic mixed signal

Assessment

hypoechoic complex fluid anechoic simple fluid soft tissue proliferation

tendon abnormality other:

Power Doppler (see last page)

1 2 3 4

Compressibility

fully partially non-compressible N/A

Patient position: supine, knee at 30° flexion

Axis: LAX

Probe position: Distal to patella

Bony landmarks: Patella (proximal) and tibia (distal) Muscles: Patellar tendon (central between patella and tibia) Blood Vessels: None

Nerves: None

Other: Hoffa fat pad inferior to patellar tendon

## Knee – Medial Meniscus and MCL

Patient name: MR number: Person completing this form: Date:

Meniscus Abnormality

tear irregularities altered echogenicity

Assessment

MCL abnormality medial meniscus abnormality irregular bony margin

other:

Power Doppler (see last page)

1 2 3 4

Compressibility

fully partially non-compressible N/A

Patient position: Supine, knee at 30° flexion, external rotation of the hip

Axis: LAX

Probe position: Medial joint space

Bony landmarks: Femur (proximal) and tibia (distal)

Muscles: None Blood Vessels: None Nerves: None

Other: Medial meniscus (between femur and tibia) and MCL (superior to meniscus)

Ankle – Anterior Joint Space SAX: *Osteochondral Line*

Patient name: MR number: Person completing this form: Date:

Joint Space Content

none anechoic hypoechoic mixed signal

Assessment

hypoechoic complex fluid anechoic simple fluid soft tissue proliferation irregular bony margin diminished joint space osteophytes

other:

Measurement

Length of osteochondral alterations:

Power Doppler (see last page)

1 2 3 4

Compressibility

fully partially non-compressible N/A

Patient position: Supine, foot rests on bolster

Axis: SAX

Probe position: Across anterior tibiotalar joint

Bony landmarks: Talus

Muscles: Superior to tibia, extensor digitorum longus tendon, extensor hallucis tendon, tibialis anterior tendon

Blood vessels: Dorsalis pedis artery and vein

Nerves: Deep peroneal nerve

Measurement (osteochondral alteration): Individually measure any variation from hyperechoic, smooth, intact bony interface. Then add the values together for the total length of alterations.

BLEED DETECTION

Ankle – Anterior Joint Space SAX: *Capsular Thickness*

Patient name: MR number: Person completing this form: Date:

Joint Space Content

none anechoic hypoechoic mixed signal

Assessment

hypoechoic complex fluid anechoic simple fluid soft tissue proliferation irregular bony margin diminished joint space osteophytes

other:

Measurement

Capsular thickness* midpoint 0.5 cm medial: 0.5 cm lateral:

Power Doppler (see last page)

1 2 3 4

Compressibility

fully partially non-compressible N/A

Patient position: Supine, foot rests on bolster

Axis: SAX

Probe position: Across anterior tibiotalar joint

Bony landmarks: Talus

Muscles: Superior to tibia, extensor digitorum longus (lateral), extensor hallucis longus (center), tibialis anterior (medial)

Blood vessels: Dorsalis pedis artery and vein

Nerves: Deep peroneal nerve

Measurement (capsular interface): Measure the thickness of the capsular interface from the beginning of the synovial membrane to the height of the dorsalis pedis artery muscle at the lowest point of the trochlea and 0.5 cm to the medial and lateral side. Assure that measurement is perpendicular to the bony margin.

*New: Capsular thickness should include all soft tissue between the synovial membrane (inferior margin) and dorsalis pedis (superior margin).

Ankle – Anterior Joint Space LAX: *Cartilage Thickness*

Patient name: MR number: Person completing this form: Date:

Joint Space Content

none anechoic hypoechoic mixed signal

Assessment

hypoechoic complex fluid anechoic simple fluid soft tissue proliferation irregular bony margin diminished joint space osteophytes

other:

Measurement

Cartilage thickness apex: 0.25 cm proximal:

Power Doppler (see last page)

1 2 3 4

Compressibility

fully partially non-compressible N/A

Patient position: Supine, foot rests on bolster

Axis: LAX

Probe position: At the midline of the anterior talocrural joint

Bony landmarks: Tibia (proximal), talar dome (center), and talar head (distal)

Muscles: Extensor digitorum longus tendon (superficial) Blood vessels: Dorsalis pedis artery (deep to tendon) Nerves: None

Other: Fat pad anterior to talocrural joint space

Measurement: Assess cartilage thickness perpendicularly to the bony margin at the apex and 0.25 cm proximally.

Ankle – Achilles Tendon: *Thickness Kager’s Fat Pad*

Patient name: MR number: Person completing this form: Date:

Assessment

abnormal tendon fibers irregular bony margin (calcaneus)

other:

Measurement

Tendon thickness 2.0 cm from calcaneus:

Power Doppler (see last page)

1 2 3 4

Compressibility

fully partially non-compressible N/A

Patient position: Prone, foot resting on a bolster

Axis: LAX

Probe position: Distal Achilles tendon

Bony landmarks: Tibia (proximal) and calcaneus (distal)

Muscles: Achilles tendon (superior), tibialis posterior tendon (superior to tibia), and flexor digitorum tendon (superior to tibialis posterior)

Blood vessels: None

Nerves: None

Other: Kager‘s fat pad (deep to Achilles tendon), retro-calcaneal bursa (between Achilles tendon and calcaneus)

Measurement (capsular interface): Assess tendon thickness by measuring perpendicularly at 2 cm proximally from the end of the calcaneus.

## Ankle – Subtalar Joint

Patient name: MR number: Person completing this form: Date:

Assessment

hypoechoic complex fluid anechoic simple fluid soft tissue proliferation

other:

Power Doppler (see last page)

1 2 3 4

Compressibility

fully partially non-compressible N/A

Patient position: Supine, foot rests on bolster, external rotation of the hip

Axis: LAX

Probe position: Connecting medial malleolus with calcaneus

Bony landmarks: Medial malleolus (proximal), sustentaculum tali (center), calcaneus (distal)

Muscles: None Blood vessels: None Nerves: None

## Impression/Assessment

Patient name: MR number: Person completing this form: Date:

Elbow:

Knee:

Ankle:

Provider signature:

## Power Doppler Score

Soft tissue perfusion is assessed by Power Doppler signals in each hemophilia joint and scored semi- quantitatively (0=no signal; 1=small spots; 2=confluent vessels in <50% tissue of interest; 3=confluent vessels in ≥ 50% tissue of interest).1, 2, 3

Depicted are scoring examples in specific joint locations. The asterisk (*) indicates effusion. Note that the PD signal acquisition rectangle is often larger than the tissue of interest, which may be a smaller area within the rectangle.

1 2 3

Elbow

Olecranon fossa

Knee

Medial meniscus

Ankle

Tibiotalar joint

1. Bhat V, et al. AJH, 2015.
2. Backhaus M. et al. Arthritis Rheum, 2009.
3. Kidder W et al. Microcirculation, 2016.
